# Escalating the conflict? Intersex genetic correlations influence adaptation to environmental change in facultatively migratory populations

**DOI:** 10.1111/eva.13368

**Published:** 2022-03-30

**Authors:** Adam Kane, Daniel Ayllón, Ronan James O’Sullivan, Philip McGinnity, Thomas Eric Reed

**Affiliations:** ^1^ School of Biology and Environmental Science and Earth Institute University College Dublin Dublin Ireland; ^2^ Faculty of Biology Department of Biodiversity, Ecology and Evolution Complutense University of Madrid (UCM) Madrid Spain; ^3^ Organismal and Evolutionary Biology Research Programme Faculty of Biological and Environmental Sciences University of Helsinki Helsinki Finland; ^4^ 8795 School of Biological, Earth and Environmental Sciences University College Cork Cork Ireland; ^5^ 8795 Environmental Research Institute University College Cork Cork Ireland; ^6^ Marine Institute Furnace Newport Ireland

**Keywords:** agent‐based models, anadromy, brown trout, genetically explicit eco‐evolutionary model, intralocus sexual conflict, sexual antagonism

## Abstract

Males and females are often subject to different and even opposing selection pressures. When a given trait has a shared genetic basis between the sexes, sexual conflict (antagonism) can arise. This can result in significant individual‐level fitness consequences that might also affect population performance, whilst anthropogenic environmental change can further exacerbate maladaptation in one or both sexes driven by sexual antagonism. Here, we develop a genetically explicit eco‐evolutionary model using an agent‐based framework to explore how a population of a facultatively migratory fish species (brown trout *Salmo trutta*) adapts to environmental change across a range of intersex genetic correlations for migration propensity, which influence the magnitude of sexual conflict. Our modelled focal trait represents a condition threshold governing whether individuals adopt a resident or anadromous (sea migration) tactic. Anadromy affords potential size‐mediated reproductive advantages to both males and females due to improved feeding opportunities at sea, but these can be undermined by high background marine mortality and survival/growth costs imposed by marine parasites (sea lice). We show that migration tactic frequency for a given set of environmental conditions is strongly influenced by the intersex genetic correlation, such that one sex can be dragged off its optimum more than the other. When this occurred in females in our model, population productivity was substantially reduced, but eco‐evolutionary outcomes were altered by allowing for sneaking behaviour in males. We discuss real‐world implications of our work given that anadromous salmonids are regularly challenged by sea lice infestations, which might act synergistically with other stressors such as climate change or fishing that impact marine performance, driving populations towards residency and potentially reduced resilience.

## 1 INTRODUCTION

Sexual conflict refers to situations where males and females have divergent evolutionary interests, leading to an evolutionary ‘tug‐of‐war’ between the sexes (Arnqvist et al., 2005). At a genomic level, intralocus sexual conflict (IASC) is where alleles influencing the same phenotypic trait(s) in each sex experience opposing selection when expressed in males versus females (Lande, 1980; Rice, 1984). The shared genetic architecture constrains independent evolution of the trait between the sexes, such that one or both get displaced from their distinct phenotypic optima (Bonduriansky & Chenoweth, 2009; Chapman et al., 2003). This generates a ‘gender load’—a reduction in mean individual fitness as a result of sexual conflict (Lande, 1980), which might increase the risk of population extirpation (Kokko & Brooks, 2003). This is especially true if there is a ‘hard’ component to selection, i.e. where the absolute fitness of an individual is set by its absolute trait value, such that variation in the strength of selection has consequences for population growth rate (Bell et al., 2021). Given the ongoing and extensive anthropogenic stressors many species are subject to in the 21st century, the fitness consequences of IASC are important to evaluate.

The presence of a gender load sets up an evolutionary pressure to ‘resolve the conflict’, potentially via modifications in genetic architecture, such as the evolution of sex‐limited or sex‐biased gene expression: two processes which erode intersex genetic correlations (Bonduriansky & Chenoweth, 2009; Lande, 1980; Rice, 1984). The breakdown of such correlations allows the trait in males and females to evolve along more independent trajectories, which can result in increased sexual dimorphism at a phenotypic level. The presence of sexual dimorphism within contemporary populations could, thus, indicate that past IASC has been resolved (Bonduriansky & Chenoweth, 2009). However, IASC is unlikely to be resolved completely across the entire genome (Bonduriansky & Chenoweth, 2009), with temporal or spatial variation in sex‐specific patterns of selection meaning that one or both sexes will not always be at their optimum, and hence, some degrees of IASC can remain. Theoretical and empirical studies also suggest that the intersex genetic correlation for sexually dimorphic traits can be maintained at nonzero values once each sex has reached their respective fitness optima (Fairbairn & Roff, 2006; Meagher, 1992; Poissant et al., 2010; Reeve & Fairbairn, 2001). Positive or negative intersex genetic correlations will then impede or accelerate sex‐specific evolutionary responses to changes in selection pressures, just as genetic covariance amongst traits expressed in the same sex can impede or accelerate adaptation (e.g. to climate change; Hellmann & Pineda‐Krch, 2007). Anthropogenic changes, in particular, can generate novel or rapidly shifting selection landscapes to which populations must adapt, move away from or else face extirpation (Carlson et al., 2014; Gomulkiewicz & Holt, 1995; Lynch & Lande, 1993; Reed et al., 2011; Vincenzi, 2014). Yet, the potential for anthropogenic change to generate new, or intensify/diminish pre‐existing sexual conflict, and the effects such changes in sexual conflict have on eco‐evolutionary dynamics, remains largely unexplored (but see Fricke et al., 2009; Kwan et al., 2008).

One key trait that may be involved in sexual conflict is migration propensity (Pearse et al., 2019). In many species, there is a gender imbalance in migration tendencies (fish: Jonsson & Jonsson, 1993; amphibians: Grayson & Wilbur, 2009; mammals: Brown et al., 1995; birds: Adriaensen & Dhondt, 1990; Palacín et al., 2009; but see Grist et al., 2017), suggesting that the fitness trade‐offs between migratory tactics may be different for males and females. Many invertebrate and vertebrate species exhibit ‘partial migration’, where populations comprise a mix of migratory and nonmigratory (resident) individuals (Chapman et al., 2011a; Fairbairn & Roff, 2006). Partial migration typically reflects conditional strategies (Lundberg, 1988), whereby individuals of a given genotype facultatively choose migration or residency depending on environmental conditions (i.e. holding genotype constant you get variation in phenotype when environmental conditions vary). Such environmental conditions may include density‐dependent factors that can vary over time (Chapman et al., 2011b; Ferguson et al., 2017; Pulido, 2011). This is adaptive when optimal tactic choice is conditional on the value of the cue. This cue could be a dynamic physiological variable such as energetic state that provides an integrated signal of recent environmental experience to the animal. The ‘environmentally cued threshold model’ (ETM) (Buoro et al., 2012; Hazel et al., 1990; Tomkins & Hazel, 2007) posits the existence of genetic variation in ‘switch‐points’, i.e. the threshold value of the status trait, above which individuals choose residency, for example, below which individuals choose to migrate. (Chapman et al., 2011b; Pulido, 2011). Crucially, the optimal switch‐point can vary not only across populations, but also within populations, and between males and females. Thus, females might be selected to choose migration at a different energy status or body size compared with males (Boyle, 2008; Grayson & Wilbur, 2009; Jahn et al., 2010; Jonsson & Jonsson, 1993; Morita et al., 2014).

Here, we explore IASC in a facultatively migratory fish that hatches in fresh water and then either remains in its natal stream for its entire life (residency tactic) or migrates to a better feeding habitat such as a larger river, lake, estuary or the open sea. We focus specifically on facultative sea migration (‘anadromy’), where fish move to the ocean to grow and return to fresh water to spawn, as an extreme form of partial migration and present a genetically explicit eco‐evolutionary, agent‐based model (ABM) framed around the ecology and life history of brown trout (*Salmo trutta*). The concepts and insights are easily generalized to facultative migration in any species where environmental changes are occurring into habitats between which individuals migrate, e.g. breeding versus wintering grounds of migrant birds. In brown trout, as in other salmonines (members of the subfamily Salmoninae: trout, charr and salmon) displaying facultative anadromy, the decision to adopt an anadromous versus stream residency life history is best explained by the ETM (Dodson et al., 2013; Ferguson et al., 2017, 2019; Jonsson & Jonsson, 1993; Kendall et al., 2014; Morita et al., 2014; Sloat et al., 2014). A decision window occurs early in life, in which individuals whose environmentally sensitive energetic status exceeds their genetically predetermined switch‐point adopt a resident tactic; if this status is not exceeded, an individual adopts an anadromous tactic (Archer et al., 2019; Eldøy et al., 2021; Ferguson et al., 2017, 2019). Fish in relatively low energy status may benefit from migrating to the sea, which is more productive than the cold, oligotrophic rivers typical of brown trout freshwater habitat. Becoming anadromous is, however, traded off against the energetic costs of migration, increased predation rates at sea and potentially increased parasitism/risk of disease at sea. In contrast, fish with a relatively high energy status can mature earlier in fresh water and avoid such costs.

Female reproductive success (in terms of both offspring quantity and quality) is more dependent on adult size than male reproductive success, and hence females benefit more from becoming anadromous (see Ferguson et al., 2019 and references therein). In contrast, male reproductive success is typically more limited by access to mates (Fleming, 1998), and males can adopt one of two mating tactics: anadromous, which comes with larger body size and hence increased ability to attract and defend females (Esteve, 2005), or sneaker, where small resident males evade competition and aggression from large anadromous males by sneaking fertilisations once the anadromous males have returned from the sea to breed in fresh water (Dellefors & Faremo, 1988; Gross, 1985). Sneaker males are found in many fish species, and negative frequency‐dependent selection (i.e. rare‐type advantage) likely plays a role in the maintenance of these alternative mating tactics (Gross, 1996; Taborsky et al., 2008). Females tend to be over‐represented amongst anadromous fish, and males amongst residents, in facultatively anadromous populations of brown trout (Ferguson et al., 2017) and other salmonines (Morita et al., 2014; Rundio et al., 2012). However, sex ratios of both tactics can vary substantially across space (Machidori, 1984; Malyutina et al., 2009; Ohms et al., 2013). This suggests that sex‐specific optimal switch‐points vary with ecological context and/or that the evolution of sexual dimorphism in switch‐points (and hence migration propensity) is constrained, in some cases, by a nonzero intersex genetic correlation for this trait, which gives rise to sexual conflict.

Using our model, we first explore scenarios in a stable environment without increased marine mortality, to illustrate how the joint evolutionary and population dynamics depend on interactions between sex‐specific selection, genetic architecture and the presence of frequency‐dependent selection on male tactics. We note that anthropogenic factors such as dams that obstruct migration within rivers (Morita et al., 2000), or fishing pressure in the marine environment (Theriault et al., 2008), can drive changes in the frequency of alternative migratory tactics (AMTs) via evolutionary responses or phenotypic plasticity (Ferguson et al., 2019). Thus, we also examine anthropogenic change in the marine environment that may alter the selective dynamics on AMT switch‐points in a sex‐specific manner—namely, increased parasite pressure. Commercial fish farms in offshore areas can lead to increased infestation rates of sea lice (*Lepeophtheirus salmonis* and *Caligus* spp.) on anadromous salmonids that migrate close to these farms, negatively impacting upon their marine growth and/or survival rates (Birkeland, 1996; Bjørn et al., 2001; Costello, 2009; Gargan et al., 2003, 2012; Martin et al., 2005; Shephard et al., 2016; Thorstad et al., 2015). We examine scenarios of environmental change driven by increased parasite pressure, to address two key questions: (1) how does genetic architecture constrain evolutionary shifts in migration propensity for each sex, and (2) how do associated changes in the intensity of sexual conflict influence population productivity?

## 2 METHODS

### 2.1 Model description

**FIGURE 1:**
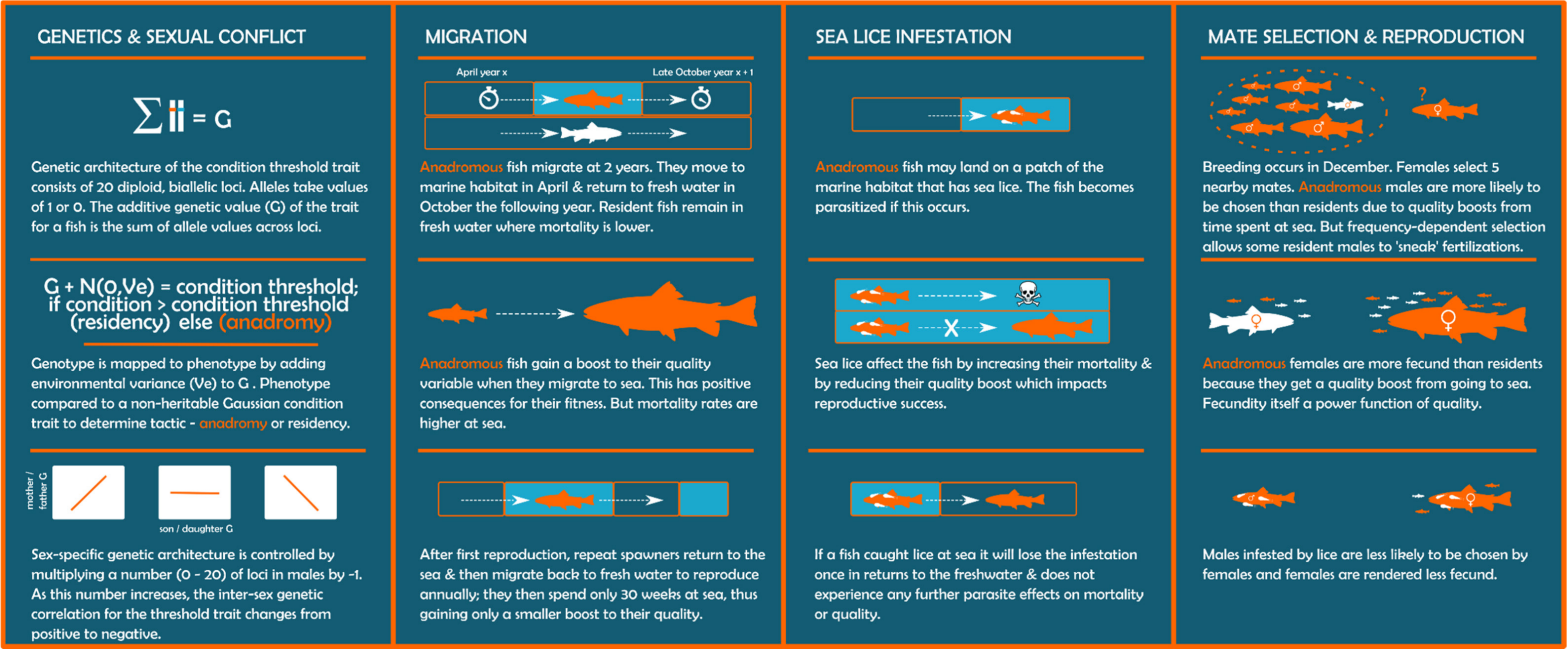
Infographic of the key components of the agent‐based model. Please refer to the ODD for further details of the modelʼs procedures

Our genetically explicit eco‐evolutionary ABM was implemented in the freely available software platform NetLogo 6.0.4 (Wilensky, 1999). We provide as Supporting Information a complete, detailed model description following the ODD (Overview, Design concepts, Details) protocol (Grimm et al., 2020) and results from a global sensitivity analysis on the modelʼs parameters (Appendix S1 and S2, respectively). Figure 1 provides a graphical description of the main facets of the model. We include below an overview of the modelʼs main features.

The model is spatially explicit and simulates the life cycle of a facultatively anadromous brown trout population using a weekly time step. Brown trout display strong life‐history variability across populations throughout their distribution (Jonsson & Jonsson, 2011), so to simplify, the model simulates the most common life history of Irish and British brown trout populations that exhibit anadromy (Solomon, 2006). Resident individuals are sexually mature in their third year of life (age‐2) and can spawn in multiple years if they survive, whilst anadromous individuals migrate to the ocean as age‐2 trout and reproduce for the first time after having spent one winter at sea (age‐3), spawning annually thereafter without further overwintering at sea (that is, repeat spawning anadromous fish return to sea in spring but migrate back to fresh water in autumn of the same year). For simplicity, individuals cannot switch tactics in the model, but we recognize that, in nature, things might be more flexible and complex (Birnie‐Gauvin et al., 2019). There are two kinds of entities: square grid cells (patches) and trout. The physical environment is evenly split into two regions representing freshwater and marine habitats, and each habitat has 5000 patches. The freshwater habitat has a carrying capacity of 3000 individuals. Trout are characterized at any given timestep by their habitat location, age, sex, migratory tactic (resident or anadromous), quality (a generic trait representing body size or mass, on which fecundity and mate choice depend), condition (a generic trait representing energy status, which is a cue for migration decisions) and the genetic material coding for the condition‐threshold trait on which the migration‐residency decision is based. Mortality and aging take place every time step, whilst all other processes occur only at one specific week of the year. The implemented biological processes, following their schedule in the model, are described next.

#### 2.1.1 Mortality

We assumed that only 10% (parameter *SurvRate*) of the eggs produced per female (section 2.1.3) survives to the fry stage (Elliott, 1994). Afterwards, surviving fish experience a density‐independent background mortality rate both in fresh water and at sea. This rate is higher at sea owing to the more hazardous environmental conditions and stresses associated with migration and life at sea, so that marine mortality for males (taken as the reference sex) is given by *mortalityM* × anad‐*death*‐*multiplierM*, where *mortalityM* is the baseline mortality of males in fresh water, and *anad*‐*death*‐*multiplierM* is a constant that represents the increased mortality experienced by anadromous males at sea (Appendix S1: Table S2). Background mortality for females is then calculated relative to males, with *mortalityF* representing a scalar that increases/decreases freshwater mortality in females relative to males, whilst *anad*‐*death*‐*multiplierF* increases/decreases marine mortality in females relative to males. Both sexes also experience a density‐dependent mortality at the fresh water when the population is over its carrying capacity. Density‐dependent mortality is mainly triggered after reproduction, so it kills off a random set of newborn individuals over the carrying capacity, as they cannot outcompete older fish. We do not include density‐dependent mortality at sea as the recruitment rate of smolts is typically below the carrying capacity in marine waters for sea trout and Atlantic salmon, so that mortality is basically driven density‐independent factors (Jonsson & Jonsson, 2011). The trout have a maximum lifespan of 8 years, an intermediate value in the range of longevities reported for the species (Jonsson & Jonsson, 2011).

#### 2.1.2 Migration

At week 14 (corresponding to early April), all age‐2 and older anadromous trout migrate to a random patch in the marine habitat. Fish at sea gain a boost to their quality (parameter *anad_quality*, which increases the quality of anadromous fish relative to a fixed baseline for residents; Appendix S1: Table S2) representing the better feeding opportunities there. Note that ‘quality’ here could represent body size, or mass, or any individual‐level, environmentally sensitive state variable influencing reproductive success (see section 2.1.3). Quality is distinct from condition, the latter being a physiological feature that is involved in defining the migratory tactics (see section 2.1.6). Anadromous trout return to the freshwater habitat at week 44 (corresponding to late October/early November) after spending two summers and one winter (first‐time spawners) or one summer (repeat spawners) in the sea. Thus, individuals can migrate more than once and accrue additional quality benefits, but the quality gained depends on the time spent at sea. No additional mortality costs were assumed for these repeat spawners.

When migrating to sea, fish can encounter a patch with sea lice and become infested. Infestation has two effects: it increases a fishʼs chances of dying whilst at sea (Skaala et al., 2014) (extramortality represented by parameter *parasite*‐*load*), and it also reduces their increase in quality (*anad_quality* is decreased by parameter *paras_quality*) such that the benefits of feeding at sea are undercut (Gjelland et al., 2014). Thus, surviving parasitised fish return to fresh water at a lower quality compared with unparasitised fish. Once parasitised fish return to fresh water, they shed all of their lice (which do not survive well in freshwater [Gjelland et al., 2014; Skaala et al., 2014]) and do not experience any further parasite effects on mortality or quality.

#### 2.1.3 Reproduction

Adults reproduce at week 48 (corresponding to late November/early December) in freshwater. Resident trout are mature in their third year of life (age‐2 trout), whilst anadromous individuals start to reproduce in their fourth year of life after spending one winter at sea (age‐3), reflecting the fact that age at sexual maturity is typically lower in resident than anadromous trout in wild populations (Jonsson & Jonsson, 2011). Females choose up to five mates from a pool of mature males within a fixed radius, selecting those with the highest quality. Anadromous males that did not pick up sea lice (unparasitised) are thus more likely to be chosen overparasitised anadromous males, who in turn are more likely than residents to be chosen. Female fecundity is an allometric function of their quality trait, so that females have gained the growth benefits of migration produce more eggs. Thus, reductions in quality due to sea‐lice infestation affect both sexes: males experience reduced mating success, whilst females experience reduced fecundity. Because age‐2 anadromous fish stay at sea for over a year before first reproduction, they miss out on mating opportunities that residents from their same cohort can avail of in the intervening year. Thus, potential reproductive benefits of anadromy are countered by later age at first reproduction.

#### 2.1.4 Sneaker behaviour

Resident males can adopt a sneaker tactic if the ratio of anadromous males relative to resident males within a fixed radius is over a threshold at the time of mating. If so, the sneaker males temporarily get a boost to their quality state variable, increasing their likelihood of becoming a mate of a female. In this way, the mating success of resident sneakers becomes higher when they are rarer. After reproduction is over, the sneaker resets its quality state variable to its initial value.

#### 2.1.5 Genetic transmission

Each new trout inherits half of their genes from their mother and half from one father randomly selected from the motherʼs mates, with equal probability of selection. We follow Piou and Prévost (2012) in using a diploid, biallelic, multilocus, genetic architecture. Twenty unlinked, functional, additive loci are modelled, where each locus can have a 1 or a 0 allele for each branch/chromosome. As such, there are three different genotypic values (0, 1, 2) possible at loci 1 through 20. The sum of these genotypic values across loci then gives an individualʼs additive genetic value, G, for our heritable condition‐threshold trait. All loci have equal weighting. Initial heritability (generation 1) on the liability scale was 0.5 in every simulation.

Sex‐specific genetic architecture is controlled by multiplying a number (0–20) of loci in males by −1. This means that as the number of loci (n‐loci) with a negative sign increases from 0 to 20, the expected value of the intersex genetic correlation for the migration propensity trait (i.e. father G vs. daughter G, or mother G vs. son G) changes from strongly positive (n‐loci = 0) to ~0 (n‐loci = 10) to strongly negative (n‐loci = 20). A positive genetic correlation would induce both sexes to evolve in the same direction and restrict evolution in different directions. A negative correlation would induce both sexes to evolve in different directions but restrict evolution in the same direction. From here on, we simply refer to this as the intersex genetic correlation but note that this always refers (unless otherwise stated) to the migration propensity trait, and not the intersex genetic correlation *for fitness*, which when negative indicates that sexual conflict is occurring (Bonduriansky & Chenoweth, 2009).

#### 2.1.6 Migratory tendency

Our model assumes a population of facultative migrants, i.e. depending on conditions, an individual trout can be anadromous or resident. The phenotype of the condition‐threshold value is modelled as the sum of its additive genetic value G and a random environmental deviation, which is drawn from a normal distribution with a mean of zero. To determine which tactic they adopt, each fish ‘compares’ its phenotypic condition‐threshold value (hereafter, simply ‘threshold’) to its actual condition, the latter assumed to be a nonheritable, sex‐specific trait (i.e. variation in condition is entirely environmentally driven, but males and females have different mean values). Note that condition, in the real world, is likely to be heritable also (Ferguson et al., 2019), but for simplicity, we ignore this possibility in our model. If the individualʼs condition exceeds its threshold, the fish becomes a resident; otherwise, it becomes anadromous. The value of the condition trait is drawn from a normal distribution of constant variance, with the mean for males set equal to the mean threshold value of males at initialization, and likewise for females. This ensures residency and anadromy are equally likely for both sexes for the starting population.

### 2.2 Model parameterization

Model parameterization (values, sources and rationale) is extensively described in the ODD document (Appendix S1). We used parameter values from the literature, using data mainly from Irish and British brown trout populations that exhibit anadromy (Appendix S1: Table S2). Model demographic outputs and proportion of trout displaying each migratory tactic were highly sensitive to the values of mortality rates in the freshwater (*mortalityM*) and marine (*anad*‐*death*‐*multiplierM)* habitats (Appendix S2); thus, these parameters were calibrated to mimic a population that has reached a stable age and phenotype structure, in which half of the individuals display an anadromous migratory tactic and half are residents, when the intersex correlation is positive (n‐loci = 0). The calibrated value of *mortalityM* was 0.01543, which represents a ~1.5% survival rate between the egg stage and seaward migration (122 weeks), within the range reported by Poole et al. (2006) and Gargan et al. (2006) (0.5–3%) for different Irish river systems. The calibrated value of *anad*‐*death*‐*multiplierM* was 1.262, which represents a 20% survival rate from the smolt stage to the return to fresh water (82 weeks), an intermediate value between the 13% reported for northern populations of one sea‐winter sea trout by Jonsson and Jonsson (2011) and that reported by Euzenat et al. (2006) for southern populations (24%). We assumed background mortality rates for males and females to be the same (i.e. *mortalityF* = 1, *anad*‐*death*‐*multiplierF* = 1). For this model application, we used body length as the ‘quality’ measure (see section 2.1.2), with resident trout attaining a size of 230 mm at reproduction (Solomon, 2006) and anadromous individuals growing 200 mm after spending 82 weeks at sea (Jonsson & Jonsson, 2011); thus, first‐time anadromous spawners reproduce at a mean size around 430 mm.

### 2.3 Simulation experiments

All experiments were carried out using NetLogoʼs BehaviourSpace, and output data were subsequently handled, plotted and analyzed in R using the *tidyverse* packages (Wickham, 2017). Each simulation lasted for 750 years; whilst this is a long‐time horizon from a management perspective, it allowed us to be sure that evolutionary equilibria had been reached or nearly reached. We collected data every simulated year on the proportion of the two migratory tactics, to examine trait (evolutionary) responses (Aim 1). To examine the consequences of trait dynamics and sexual conflict for population dynamics (Aim 2), we counted the total number of eggs produced by all spawners in that year as well as population productivity (number of eggs divided by number of spawning females per year). We focus on females here, who are in the ‘demographic driving seat’, i.e. female reproductive success is not limited by the number of males. To compare outcomes from different simulation experiments, we calculated the median of both metrics across the final 150‐year period (i.e. after 600 years had elapsed and evolutionary equilibrium was reached or nearly reached). Each experiment was replicated 50 times unless otherwise stated.

#### 2.3.1 Experiment 1: Default scenarios with no sea lice and higher background mortality at sea than in fresh water

Our baseline scenario considered cases where there were no sea lice, but background mortality experienced by the anadromous tactic at sea was still higher than that in fresh water, in both males and females (*anad*‐*death*‐*multiplierM* = 1.264 = *anad*‐*death*‐*multiplierF*). We simulated the eco‐evolutionary trajectory of the population under three intersex genetic correlations—strongly positive, zero and strongly negative (i.e. n‐loci = 0, 10 and 20 respectively)—and allowed for the sneaker tactic to occur in each case (i.e. 3 scenarios in total). This experiment was run to understand the effect of sexual conflict on the evolution of migratory tactic frequencies under a baseline scenario, in which quality gains at sea are traded off with higher mortality risk.

#### 2.3.2 Experiment 2: Scenarios with no sea lice and equal background mortality at sea and in fresh water

This experiment was designed to explore how changes in the intersex genetic correlations (i.e. different n‐loci values) affect sex‐specific evolutionary responses to changes in selection pressures driven by improved survival at sea for both sexes relative to the baseline scenario (Experiment 1). On top of this, Experiment 2 also explored the role of the sneaker behaviour in the maintenance of the alternative migratory tactics. We ran scenarios where there was no additional mortality cost for males or females of going to sea (resident mortality = anadromous mortality, i.e. *anad*‐*death*‐*multiplierM* = anad‐*death*‐*multiplierF* = 1). Thus, anadromy might be favoured in these scenarios if the growth benefits of going to sea are sufficient to outweigh costs associated with later maturity, but this might only be true for females, who benefit more in reproductive success terms from larger size (quality, in our model) than males. This experiment was set without any sea lice, tested across intersex genetic correlation values from positive to negative (n‐loci from 0 to 20 in jumps of 2, i.e. 11 scenarios), and with the sneaker tactic either turned on or off (11 × 2 = 22 scenarios).

#### 2.3.3 Experiment 3: Scenarios with no sea lice; equal background mortality at sea and in fresh water for males, but marine mortality for females increased across different runs

This experiment explored sex differences in life‐history trade‐offs (survival versus growth) influencing the evolution of migratory tactic frequencies. We kept mortality at sea for males equal to their freshwater mortality (*anad*‐*death*‐*multiplierM* = 1) but sequentially increased (across different runs) mortality for females at sea relative to background mortality in freshwater (eight values of *anad*‐*death*‐*multiplierF* explored, ranging from 1.1 to 2.0). This would reflect known instances where females migrate earlier to sea (Berg & Berg, 1989), spend longer at sea (Berg & Berg, 1989; Jensen, 1968) or migrate further from the river and to riskier habitats (Bordeleau et al., 2018) than males. All mortality parameters were constant within runs for each scenario. Again, this was carried out without any sea lice and tested across positive, zero and negative values for intersex genetic correlation (n‐loci = 0, 10 and 20 respectively) with the sneaker tactic always on (24 scenarios in total).

#### 2.3.4 Experiment 4: Scenarios with sea lice

To examine the effects of sea‐lice infestation, we ran a full factorial design that accounted for the three factors controlling the impact of parasitism: (1) The proportion of marine habitat patches with sea lice (*prop*‐*parasites*), which was varied over three levels: 0.1, 0.4 and 0.6. These correspond to chances of sea trout landing on a patch with parasites of 10%, 40% and 60% respectively. (2) The effect of sea lice on quality (i.e. the proportion of quality gained at sea that is reduced by sea‐lice infestation; *paras_quality*), which was varied over two levels: 0.4 (i.e. 40% reduction) and 0.8 (80% reduction)—both within the range of values reported in observational and simulation studies (Halttunen et al., 2018). (3) The effect of sea lice on mortality (*parasite*‐*load*), which was varied over three levels: 1 (no extra mortality), 1.25 (25% increased mortality) and 1.6 (60% increased mortality), covering the range of values reported in natural and experimental conditions (Thorstad et al., 2015), and in modelling studies (Hedger et al., 2021). This experiment was run across three intersex genetic correlations (n‐loci = 0, 10 and 20) with the sneaker tactic always on 3 × 2 × 3 × 3 = 54 scenarios.

## 3 RESULTS

### 3.1 Experiment 1

**FIGURE 2:**
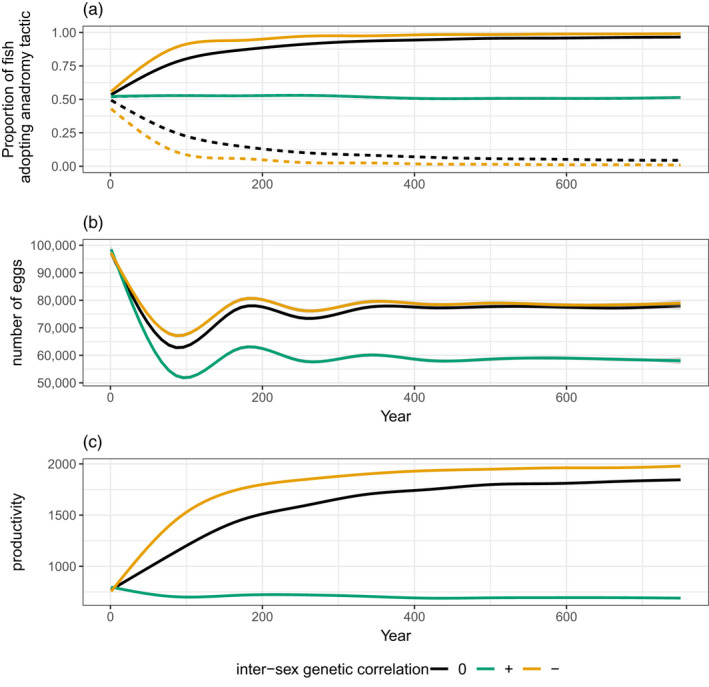
(a) Evolutionary trajectory of migratory tactic frequencies over time for Experiment 1 (baseline scenarios), where the proportion of residents = 1—proportion of anadromous fish. Continuous lines = females; dashed lines = males. (b) trajectories of number of eggs over time. (c) trajectories of the population productivity over time for Experiment 1. Productivity is the number of eggs divided by the number of spawning females per year. Note that for b and c, the male line is masked by the female line, as they exactly track each other

The sign and magnitude of the intersex genetic correlation had a strong sex‐specific effect on migratory tactic frequencies and population productivity (Figure 2). When this correlation was strongly positive, no directional evolution occurred in either males or females, who both remained at their initial starting value of equal proportions of residents and anadromous fish. When the intersex genetic correlation was close to zero (both sexes free to evolve independently), males tended towards residency and females to anadromy. When the intersex genetic correlation was strongly negative, this pattern was amplified and accelerated such that males became nearly entirely resident and females entirely anadromous within our 750‐year time horizon. There was also a 35% increase in the median number of eggs from the strongly positive to the strongly negative intersex genetic correlation scenario (Figure 2b). The difference in final egg numbers (i.e. median over the last 150 years) was minor between the n‐loci = 10 and n‐loci = 20 scenarios. The productivity of the population closely mapped to the trajectory of the frequency of anadromous females in the population over time (Figure 2a vs Figure 2c).

### 3.2 Experiment 2

**FIGURE 3:**
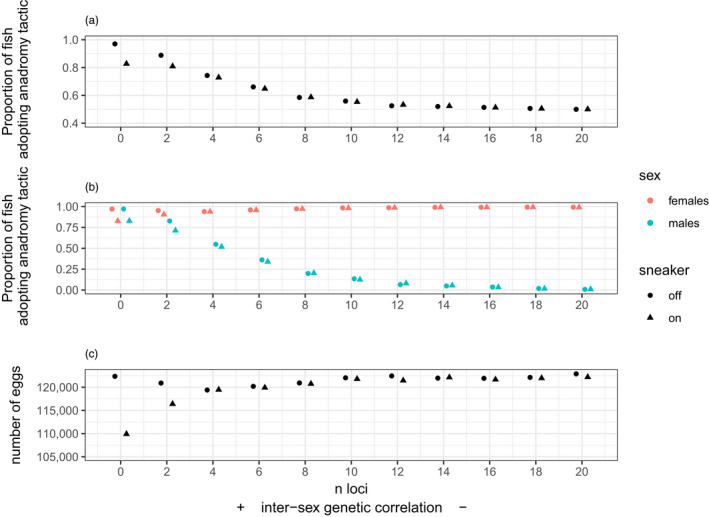
Results from Experiment 2 showing the median proportion of the anadromous tactic in the population as a whole (a); the median proportion broken down by sex (b); and the median number of eggs (c). These medians are calculated across 150 years after 600 years have elapsed and are plotted against 11 values of the intersex genetic correlation. Shown are the results for cases when the sneaker tactic is on(triangles) or off (circles)

In this experiment, there was no survival cost of going to sea, only a reproductive benefit (mediated by quality) that was weighed against a cost in terms of later age at first maturity for anadromous migrants. When both sexes were free to evolve independently (intersex genetic correlation ~0; n‐loci = 10), females tended towards complete anadromy and males towards complete residency in this experiment (Figure 3). Whilst females reached 100% anadromy after 750 years, males did not quite reach 100% residency (Figure 3) when n‐loci was 10. Longer runs of this independent evolution scenario showed that males eventually reach full residency (Figure S1B). The rate at which the sexes evolved towards their respective different optima was accelerated when the intersex genetic correlation was strongly negative (n‐loci values towards 20) because of correlated evolution in males and females that facilitates, rather than impedes, adaptation of each sex. In contrast, when the intersex genetic correlation was strongly positive (n‐loci values closer to 0), adaptation in one sex dragged the other away from its selective optimum and hence both ended up maladapted. Interestingly, both sexes equilibrate at an anadromy proportion of ~0.8 in the n‐loci = 0 scenario, indicating that a strongly positive intersex genetic correlation pulls males away from their optimum more than it does females from theirs. We can also see an effect of sneaking behaviour emerging when the correlation is strongly positive. In these scenarios, the predominant adoption of anadromy by males makes sneaking a viable tactic, thus impeding the evolution towards complete anadromy in both sexes (compare triangles to circles in Figure 3). The reduction in the proportion of anadromous females, when n‐loci = 0, means that the median number of eggs is diminished by about 10% compared to scenarios where there is no sneaker option (Figure 3c).

### 3.3 Experiment 3

**FIGURE 4:**
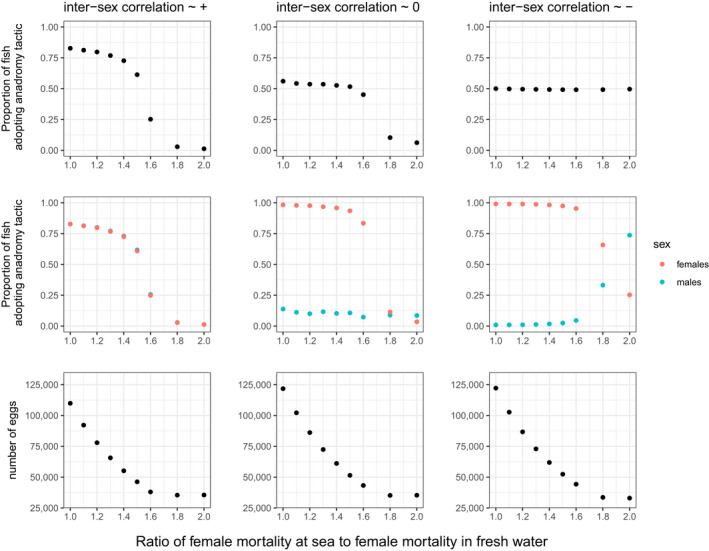
Results from Experiment 3 showing the median proportion of the anadromous tactic in the population as a whole (top); the median proportion broken down by sex (middle); and the median number of eggs (bottom). These medians are calculated across 150 years after 600 years have elapsed and are plotted against the ratio of female mortality at sea to female mortality in fresh water. Each column shows a different intersex genetic correlation

As the survival cost of anadromy increased for females, they tended towards residency, and this tendency accelerated after crossing a tipping point whose value was modulated by the intersex genetic correlation (Figure 4). There was a strong effect of varying the intersex genetic correlation; with a negative correlation, the shift in females towards adopting a resident tactic at very high marine mortality values dragged males off their optimum and drove them towards anadromy, whereas this did not occur under a zero correlation (middle panels, Figure 4). Under a positive correlation, the femaleʼs tendency towards residency at very high marine mortality values drove males in the same direction and thus towards their optimum (left panels, Figure 4).

As mortality of females at sea was increased, egg production diminished, but the rate of decline levelled off when the ratio of female mortality at sea to female mortality in fresh water exceeded ~1.6 (bottom panels, Figure 4). This was the region of parameter space where the proportion of anadromous females started to drop much more rapidly (middle panels, Figure 4), so once females had become mostly resident, further increases in female marine mortality were of no demographic consequence. Changes in egg production were little affected by the intersex genetic correlation, as changes in the proportion of anadromous females were similar in each case—at least when the correlation was zero or positive. With a negative intersex genetic correlation, females did not evolve towards complete residency when female marine mortality was at its highest relative to freshwater mortality, yet egg production fell to a similar value when the correlation was zero or positive (bottom right panel, Figure 4). This seemed to reflect hidden demographic impacts of sexual conflict because both sexes (especially males) were dragged away from the male–female joint optimum (full residency) when the intersex genetic correlation was negative (middle right panel, Figure 4). Thus, females still exhibited some anadromy (~25%) at the highest level of female marine mortality, which would come with a survival cost, but this appeared to be offset by their increased fecundity relative to residents and an increase in the number of spawners overall (Figures S2 andS3).

### 3.4 Experiment 4

**FIGURE 5:**
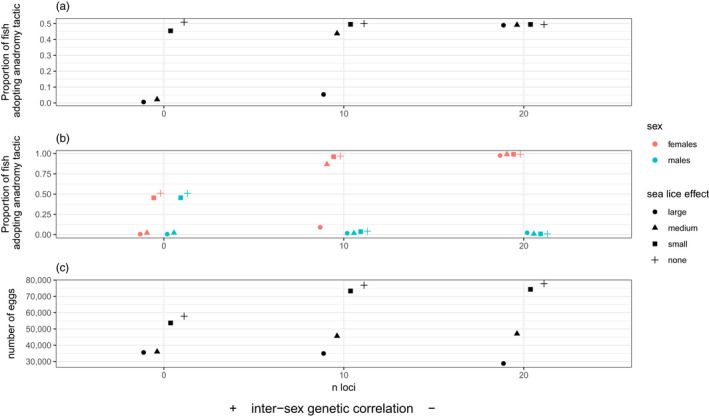
Results from Experiment 4 where the effects of sea‐lice infestation were explored. Results show the median tactic proportions (both sexes taken together) in the population across 150 years after 600 years have elapsed (a) and split by sex (b). Bottom panel shows the median number of eggs produced, again after 600 years has elapsed (c). Shapes correspond to different intensities of the effect of sea‐lice infestation. Effects were tested across three levels of intersex genetic correlation. Small sea‐lice effects correspond to prop‐parasites = 0.1, parasite‐load = 1 and paras_quality = 0.4; medium sea‐lice effects correspond to prop‐parasites = 0.4, parasite‐load = 1.6 and paras_quality = 0.4; large sea‐lice effects correspond to prop‐parasites = 0.6, parasite‐load = 1.6 and paras_quality = 0.8. Refer to section 2.3.4 for more information

Sea‐lice infestation had strong sex‐specific effects on migratory tactic frequencies that were modulated by the intersex genetic correlation (Figure 5 shows three examples relative to the baseline where sea‐lice effects were small, medium or large). Overall, sea lice shifted proportions towards residency relative to the baseline. When the effects of sea lice were low, sex‐specific evolutionary trajectories did not differ markedly from the baseline scenario without sea lice. Thus, females were selected towards anadromy and males towards residency, and resulting evolution was accelerated by a negative intersex genetic correlation (because selection was of opposite sign in each sex) and impeded by a positive intersex genetic correlation. When the effects of sea lice increased to moderate levels, anadromy still appeared to be the optimal tactic for females, and residency for males, when the sexes were free to evolve independently (n‐loci = 10; intersex genetic correlation ~0). However, both males and females evolved to near‐complete residency under the strong positive intersex genetic correlation scenario (n‐loci value = 0). Only under scenarios of extreme effects of sea lice did females evolve to residency when they were evolutionarily unconstrained (intersex genetic correlation ~0), but they were dragged off this optimum by males being selected towards complete residency when the intersex genetic correlation was strongly negative (n‐loci value = 20).

Sea‐lice infestation decreased egg production (Figure 5 bottom panel). Varying the intersex genetic correlation modulated the impact under the scenarios where sea‐lice effects were most extreme (high proportion of marine patches with lice, high lice load and high lice effect on quality), such that egg production declined as the intersex genetic correlation varied from positive to zero to negative, because of the strong decline in the number of female spawners (Figures S4–S6).

## 4 DISCUSSION

### 4.1 Sexual antagonism pulls one or both sexes off their environmental optimum

Our results show that sexual conflict can emerge under certain combinations of environmental conditions and genetic architectures and have a substantial effect on the evolution of migratory tactics. This, in turn, can have repercussions for population demography—particularly when females are dragged off their selective optimum—emphasizing how understanding sexual conflict is of applied relevance (e.g. in fisheries management or conservation biology), as well as being of intrinsic interest. When males and females are selected in different directions, a positive intersex genetic correlation for the selected trait can prevent either or both sexes from evolving to their optimum. Conversely, when males and females are selected in the same direction, a negative intersex genetic correlation for the trait constrains sex‐specific adaptation. If the absolute strength of selection on each sex is similar, a ‘stalemate’ can occur such that both ‘lose’ (i.e. end up maladapted), as occurred in our model under Experiment 1 when the intersex genetic correlation for migration propensity was strongly positive. Under that set of environmental conditions, full residency was optimal in males and full anadromy optimal in females, yet both sexes remained evolutionarily ‘stuck’ with intermediate tactic frequencies (Figure 2).

**FIGURE 6:**
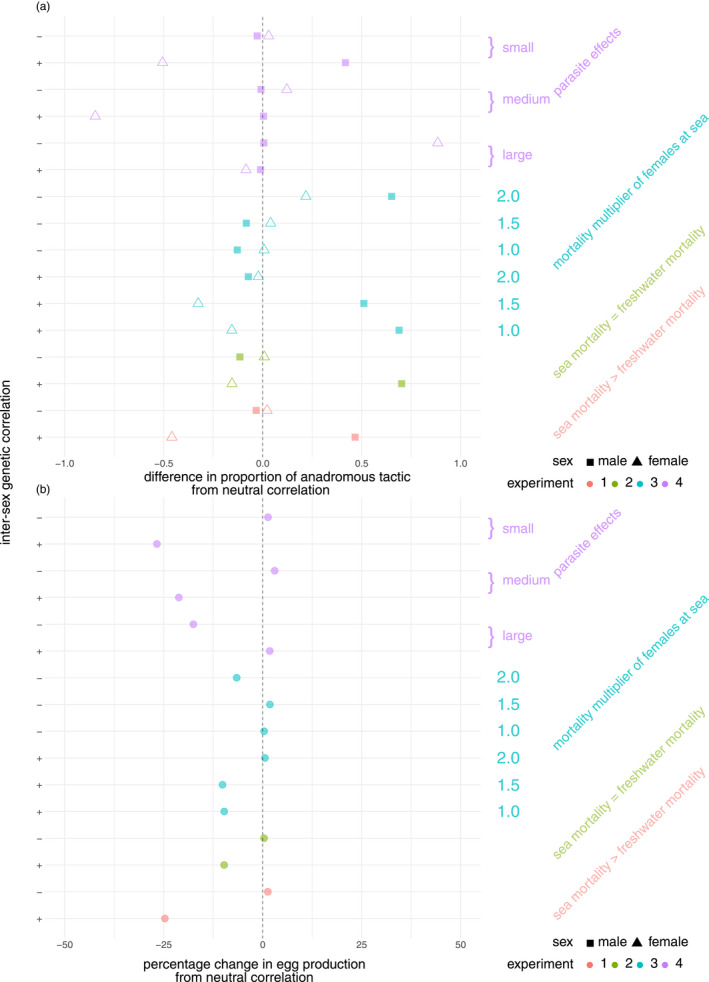
Sample of simulations across experiments to show the extent of maladaptation relative to a baseline where there is a zero intersex genetic correlation. (a) shows how the sex‐specific anadromy proportions differ from a neutral correlation. (b) shows the percentage change in median total egg number from a neutral correlation

When selection is stronger in absolute terms in one sex, this sets up an asymmetry such that one sex is dragged off its optimum more (i.e. ‘loses’ worse) than the other sex (Plesnar‐Bielak & Łukasiewicz, 2021). Such asymmetric outcomes occurred in our model under experiments 2–4. In experiment 2, males ‘lost’ (were pulled towards anadromy) when the intersex genetic correlation was strongly positive (Figure 3b). Males also fared worse in experiment 3 (again, were pulled away from full residency) when the intersex genetic correlation was strongly negative and marine mortality for females was high and also when intersex genetic correlation was strongly positive and marine mortality for females was below the ‘shift‐threshold’. (Figure 4b). In experiment 4, a strongly positive intersex genetic correlation kept both sexes off their optima (full residency for males and full anadromy for females) by similar amounts when the effects of sea lice were low, but when sea‐lice effects were high, a negative genetic correlation pulled females off their optimum (i.e. away from full residency) but not males (Figure 5b). The more females were driven off their environmental optimum by sexual antagonism in any given experiment, the greater the resulting demographic impacts at the population level, which involved either reductions in number of spawning females, per‐female fecundity or both—and hence total egg production (Figure 6; Figure S7). In contrast, sexual‐antagonism‐induced maladaptation in males had much weaker demographic consequences, although sneaking behaviour in males (when ‘on’ in the model) could have indirect demographic consequences, e.g. by preventing females from reaching full anadromy under a strongly positive intersex genetic correlation (Figure 3c).

### 4.2 Sexual antagonism can maintain a mix of tactics in one or both sexes despite environmental change

In our baseline scenario (Experiment 1), females evolved to complete anadromy and males to complete residency in the absence of genetic constraints, i.e. when the intersex genetic correlation was close to zero (Figure 2a, black line). In many real‐world salmonine populations, of course, both sexes can exhibit a mix of resident and migratory tactics, so various balancing selection mechanisms must be at play (Ferguson et al., 2017, 2019; Kendall et al., 2014; Morita et al., 2014). Sexual antagonism is one such mechanism but has hitherto received little attention. A positive intersex genetic correlation in the baseline scenario, for example, maintained an approximately equal ratio of residents to anadromous migrants in both sexes (Figure 2a, green line). This baseline scenario also served as a useful benchmark against which to judge how environmental change alters eco‐evolutionary outcomes. For instance, males were still selected towards residency when free to evolve even when there was no excess mortality suffered by fish adopting the anadromous tactic (Figure 3b), as might occur if marine conditions improved under certain climate regimes (Beamish & Bouillon, 1993), or harvest pressures on anadromous individuals were reduced (Theriault et al., 2008). Yet, males evolved towards high (but not necessarily 100%) rates of anadromy under scenarios with strongly positive intersex genetic correlations (Figure 3b), resulting in the observed sexual antagonism.

As the ratio of female mortality at sea to that in fresh water increased, the benefits of females adopting the anadromous tactic reduced to the point that they evolved to (near full) residency, like males, in the absence of any intersex genetic correlation (Figure 4b). But when the female mortality ratio was high and the intersex genetic correlation was negative, evolutionary responses in females dragged males substantially towards (maladaptive) anadromy, whilst males dragged females only a little towards anadromy (Figure 4b, right column). Conversely, when the female mortality ratio was low, a positive intersex correlation pulled males much more towards maladaptive anadromy, whilst females were pulled only a little towards maladaptive residency (Figure 4b, left column). Thus, absolute strength of selection in experiment 3 appeared to be stronger in females than in males, resulting in asymmetric evolutionary outcomes.

In experiment 4, when free to evolve, males were always selected towards residency regardless of the magnitude of sea‐lice effects, whilst females were selected towards anadromy when sea‐lice effects were small or medium, but towards residency when sea‐lice effects were large (Figure 5b, intersex correlation ~0, i.e. n‐loci = 10). Again, however, the sign of the genetic correlation had a strong effect on evolutionary outcomes. A strongly positive correlation saw both sexes shift from roughly 50:50 residents:anadromous when sea‐lice effects were small to evolve a purely resident tactic when sea‐lice effects were medium to large (Figure 5b, n‐loci = 0). With a strongly negative intersex correlation, females evolved to complete anadromy and males to complete residency regardless of the magnitude of sea‐lice effects (Figure 5b, n‐loci = 20). Thus, sex‐specific evolutionary responses to changes in marine parasite pressure were strongly affected by sex‐specific genetic architecture, and similar outcomes might be expected under other types of anthropogenic pressures such as harvest (Theriault et al., 2008) or climate change. Moreover, marine and freshwater stressors might interact in complex ways to drive life history and demographic changes in salmonine populations (Cline et al., 2019; Piou et al., 2015; Vollset, 2019), with sexual antagonism adding an additional (interesting) layer of complexity.

### 4.3 Sneaking behaviour in males can further modulate eco‐evolutionary outcomes

Recent work on sneaker males in Sockeye salmon (*Oncorhynchus nerka*) in Alaska demonstrated that the dynamics of alternative male mating tactics can have important ramifications for the productivity of exploited populations, where natural temporal variation in recruitment, coupled with size‐selective harvest, disrupts the stabilizing influence of frequency‐dependent selection (DeFilippo et al., 2019). We saw the clearest expression of sneaker effects in Experiment 2 where there was no additional mortality cost of adopting the anadromous tactic (Figure 3). When the intersex genetic correlation was positive, females pulled males off their optimum resulting in more anadromous males than the zero correlation scenario. This meant resident males were regularly surrounded by enough anadromous rivals to meet the threshold for sneaking. Sneaking being a viable tactic prevented males from reaching full anadromy, which, in turn, impeded females from reaching full anadromy. This resulted in a decline in median egg production. Yet, as the intersex genetic correlation changed from positive to negative, the increased tendency of males to evolve towards residency did not trigger the sneaking behaviour (as the sneaking threshold was no longer met) and egg production increased. Thus, the expression of sneaking, which is a function of the frequency of anadromous males, itself can change as a function of the costs of anadromy and the sign of the intersex genetic correlation.

### 4.4 Maladaptation in females owing to sexual antagonism negatively affects population productivity

In experiments without sea lice, substantial reductions in productivity were observed whenever females were dragged off their optimum by sexual antagonism (Figure 6). As the costs of anadromy for females increased, total egg production steadily declined in absolute terms (Figure 4), but when these costs were low, egg production was slightly lower in relative terms when sexual conflict dragged females towards residency (Figure 4, compare left with central column). In general, sea lice reduced total egg production through effects on female mortality and quality, which have been documented in empirical studies (Jackson et al., 2011). In the Burrishoole system in the west of Ireland, a collapse in the anadromous component of the brown trout population in the early 1990s coincided with sea‐lice outbreaks following the establishment of near‐shore Atlantic salmon (*Salmo salar*) fish farms (Poole et al., 2006). Our results illustrate not only how rapid sea‐lice‐induced evolution towards residency could be one explanation for such collapses, but also that sexual antagonism can maintain some anadromous females. In our large sea‐lice effects scenario, for example, females actually evolved to near‐complete anadromy, when the intersex genetic correlation was strongly negative (Figure 5). Yet, somewhat paradoxically, total egg production was even lower, then, compared to when the intersex correlation was zero and females were free to evolve towards residency, because maladaptation in females reduced the number of female spawners (Figure S7). Thus, the same environmental stressor could have radically different effects on a population owing to different signs of the intersex genetic correlation.

### 4.5 Limitations of our model and future opportunities

As with any model, it was necessary to make simplifying assumptions in order to limit model complexity and therefore keep things tractable, but future studies could relax/change some of these assumptions to increase realism, broaden the scope or tailor the model to specific management or conservation issues of interest. For example, we did not allow for any variation amongst anadromous fish in time spent at sea, nor variation in age at smolting, both of which might impact the cost‐benefit calculus of migration with possible demographic ramifications. Our modelʼs genetic architecture was simple with 20 unlinked, additive loci affecting a single trait without mutation. More complex architectures could be modelled, including pleiotropy (Jager, 2005), epistasis (Marty et al., 2015) and large‐effect loci (see section 7.9 in Appendix S1). Increasing numbers of studies are finding large‐effect loci or supergenes that account for a substantial fraction of intrapopulation life‐history variation (e.g. Barson et al., 2015; Kuparinen & Hutchings, 2019), with evidence in rainbow trout (*Oncorhynchus mykiss*) that sex‐specific migratory propensities are controlled by a large (55 Mb) double inversion that also exhibits sex‐dependent dominance, which might help to resolve sexual conflict (Pearse et al., 2019). Eco‐evolutionary outcomes can be less predictable with monogenic/oligogenic architectures compared to polygenic architectures (Kuparinen & Hutchings, 2017, 2019; Oomen et al., 2020), and the latter can also confer higher population viability under environmental change (Kardos & Luikart, 2021).

The nature of environmental change can also influence the dynamics, intensity and demographic impacts of sexual conflict (Plesnar‐Bielak & Łukasiewicz, 2021). Whilst conflict can be resolved in a stable environment by various genomic mechanisms (Bonduriansky & Chenoweth, 2009), it remains commonplace across a diversity of species (Van Doorn, 2009) and can re‐emerge in novel ecological contexts (Plesnar‐Bielak & Łukasiewicz, 2021), and hence, it might be a strong force‐shaping eco‐evolutionary responses in the Anthropocene. Gene flow in spatially structured populations (e.g. salmonids) might further influence both the extent of sexual conflict and its demographic impacts (e.g. Harts et al., 2014), which might be factors worth considering in supplementation programmes (Fraser, 2008) or assisted migration strategies (Hewitt et al., 2011).

In conclusion, our model draws attention to how sexual conflict—an often overlooked but crucial factor influencing evolutionary dynamics—can strongly influence patterns of life‐history evolution in response to anthropogenic change, with concomitant demographic consequences of relevance to wildlife management and conservation. In the specific context of salmonid biology, we emphasize how increased marine mortality related to sea lice represents a localized but strong selective pressure that might act in tandem with other stressors such as fishing or climate change to drive populations towards increased residency and hence have economic (e.g. reduced commercial, recreational or artisanal fishing opportunities) as well as ecological ramifications.

## CONFLICT OF INTEREST

The authors declare that they have no conflicts of interest with the work herein.

## Supporting information

Fig S1‐S7

Appendix S1

Appendix S2

## Data Availability

Since this is a simulation study that produces anticipatory predictions, no new data were generated during the preparation of this manuscript. Code for the model, initial parameter values and details of experimental settings are available on GitHub (https://github.com/kanead/trout‐migration‐abm) and in the [Link], [Link], [Link] available online.
