# Supplementary material for: Escalating the conflict? Intersex genetic correlations influence adaptation to environmental change in facultatively migratory populations
Source: Evol Appl. 2022 Mar 30;15(5):773–89. doi: 10.1111/eva.13368 (PMC9108303; doi:10.1111/eva.13368)
Supplement: Supplementary file 1 — Fig S1‐S7 [file EVA-15-773-s002.docx]

**Supplementary Material –**

**Escalating the conflict? Inter-sex genetic correlations influence adaptation to environmental change in facultatively migratory populations.**

*Experiment 2: Scenarios with no sea lice and equal background mortality at sea and in fresh water*

*2.1: long term experiment*


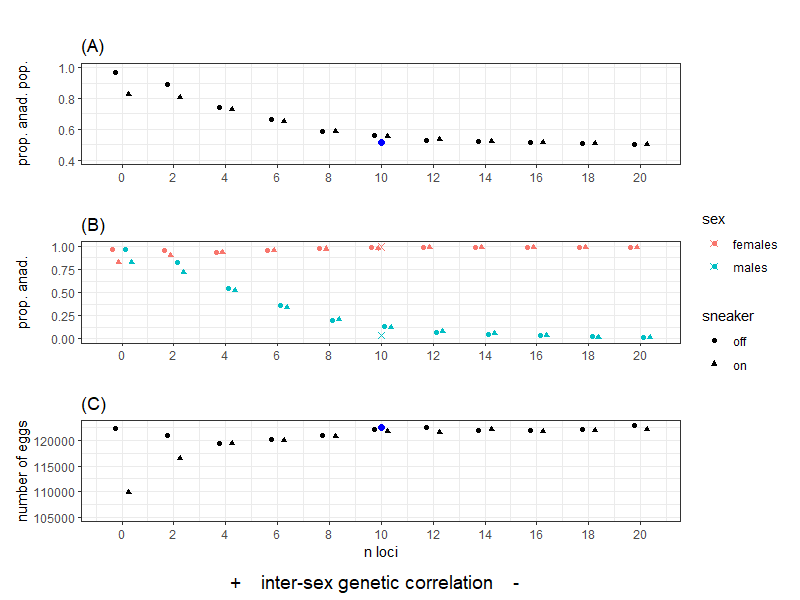


Figure S1 – Modified version of Figure 2 from Experiment 2 showing the median proportion of the anadromous tactic in the population as a whole (A); the median proportion broken down by sex (B); and the median number of eggs (C). These medians are calculated after 600 years have elapsed and are plotted against 11 values of the inter-sex genetic correlation. Shown are the results for cases when the sneaker tactic is on or off. In addition, the median results of the last 150 years of long-term experiment (5000 years) when sneaker is on and n loci = 10 are also displayed in blue (A) and (C) and crosses (B).

*Experiment 3: Scenarios with no sea lice; equal background mortality at sea and in fresh water for males, but marine mortality for females increased across runs.*


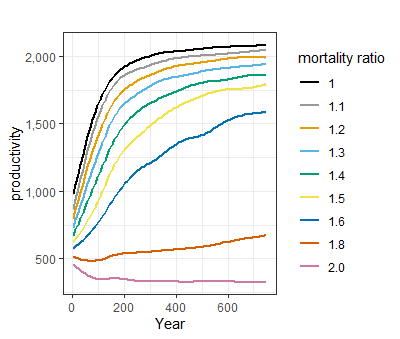


Figure S2 - Trends in productivity across 9 values of the ratio of female mortality at sea to female mortality in fresh water. This is based on n loci = 20 i.e., a negative inter-sex genetic correlation.


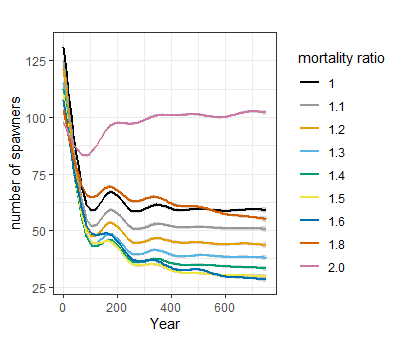


Figure S3 - Trends in the number of spawners across 9 values of the ratio of female mortality at sea to female mortality in fresh water. This is based on n loci = 20 i.e., a negative inter-sex genetic correlation.

*Experiment 4: Scenarios with sea lice*


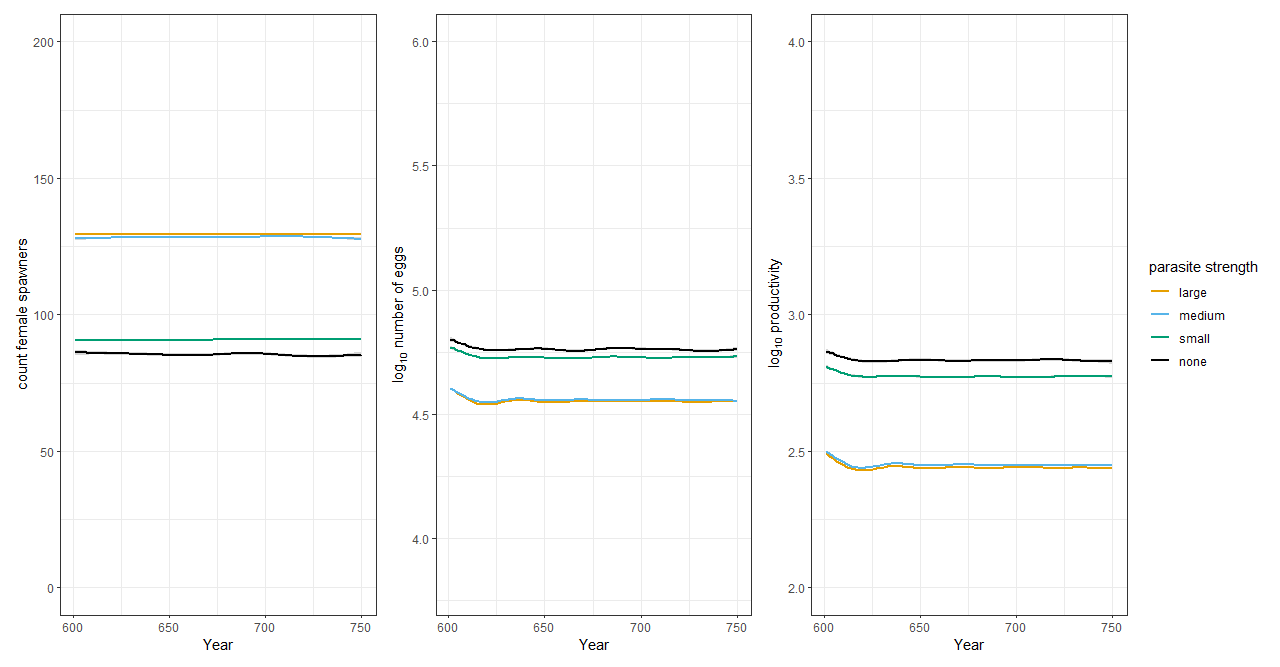


Figure S4 – Time series of the median number of female spawners across replicates (left); the log_10_ median number of eggs across replicates (middle); the log_10_ median productivity which is calculated as the number of eggs divided by the number of spawning females each year. These plots are based on n loci = 0 i.e., a positive inter-sex genetic correlation and across increased effects of parasitism.


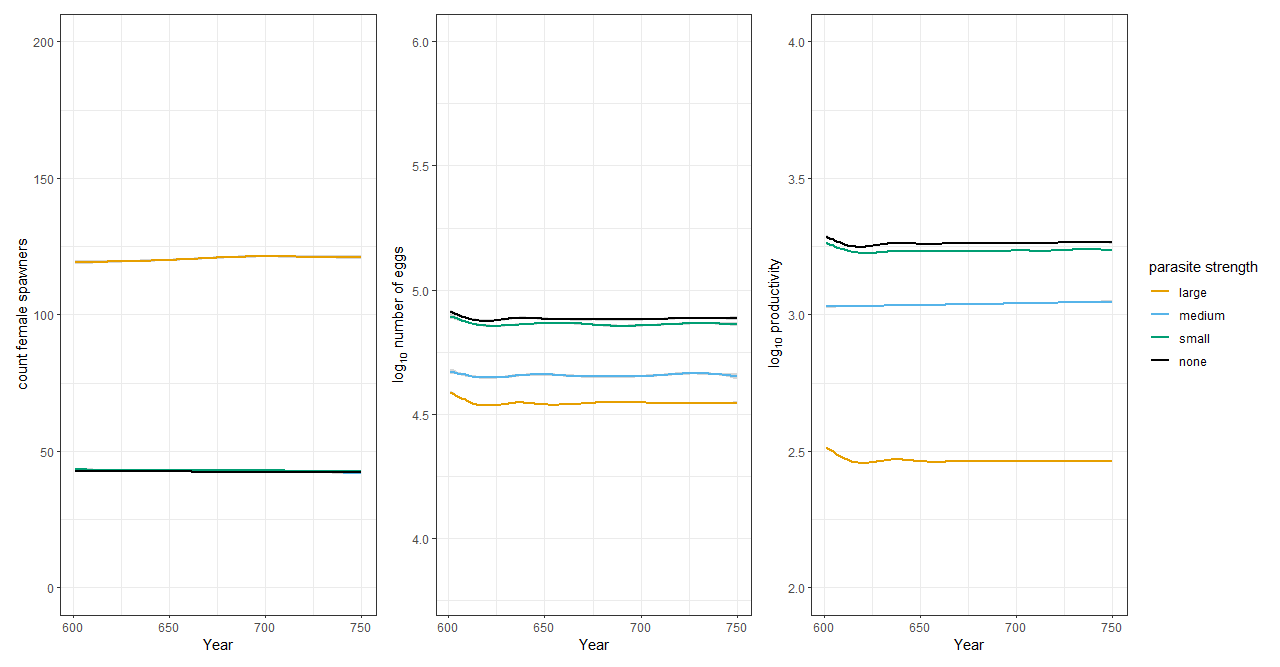


Figure S5 - Time series of the median number of female spawners across replicates (left); the log_10_ median number of eggs across replicates (middle); the log_10_ median productivity which is calculated as the number of eggs divided by the number of spawning females each year. These plots are based on n loci = 10 i.e., a neutral inter-sex genetic correlation and across increased effects of parasitism.


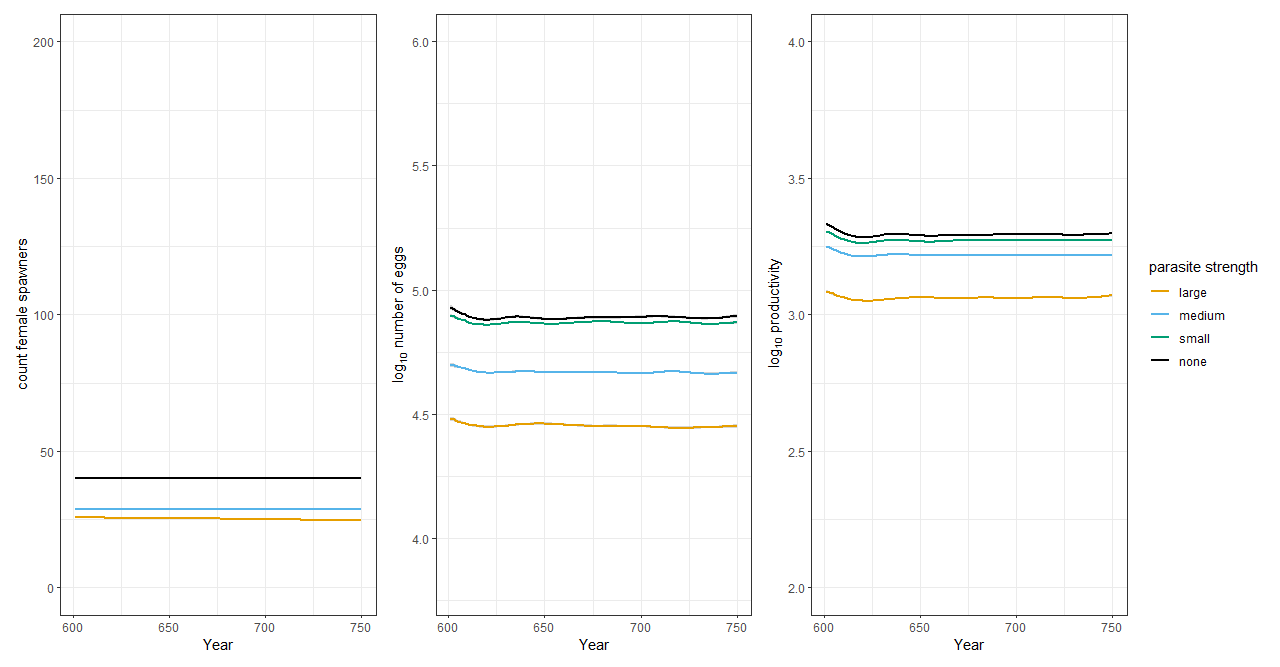


Figure S6 - Time series of the median number of female spawners across replicates (left); the log_10_ median number of eggs across replicates (middle); the log_10_ median productivity which is calculated as the number of eggs divided by the number of spawning females each year. These plots are based on n loci = 20 i.e., a negative inter-sex genetic correlation and across increased effects of parasitism.


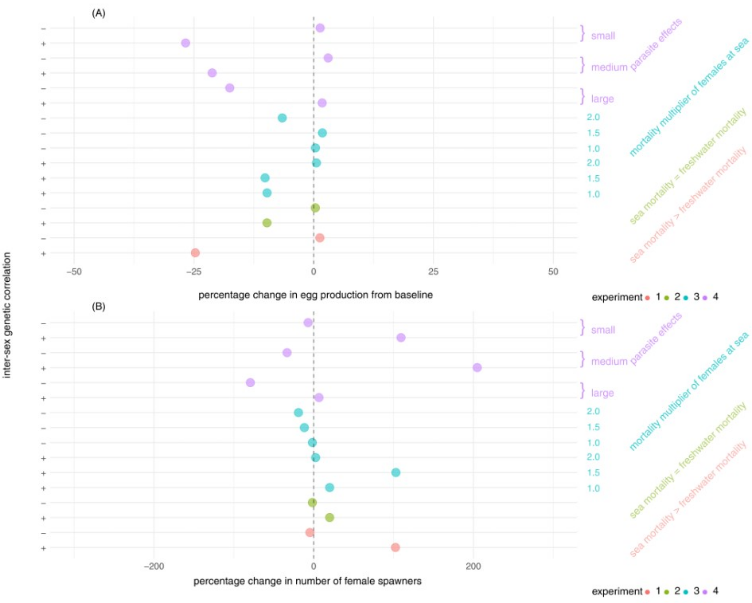


Figure S7 - Sample of simulations across experiments to show the extent of maladaptation relative to a baseline where there is a neutral inter-sex genetic correlation. (A) shows the percentage change in egg production from baseline. (B) shows the percentage change in the number of female spawners from the baseline.
