# Supplementary material for: Escalating the conflict? Intersex genetic correlations influence adaptation to environmental change in facultatively migratory populations
Source: Evol Appl. 2022 Mar 30;15(5):773–89. doi: 10.1111/eva.13368 (PMC9108303; doi:10.1111/eva.13368)
Supplement: Supplementary file 2 — Appendix S1 [file EVA-15-773-s003.docx]

**Supplementary material:**

# Sea Trout Model description

[1 Purpose 1](#_Toc412759132)

[2 Entities, state variables, and scales 1](#_Toc412759133)

[3 Process overview and scheduling 3](#_Toc412759134)

[4 Design concepts 4](#_Toc412759135)

[5 Initialization 6](#_Toc412759136)

[6 Input data 7](#_Toc412759137)

[7 Submodels 7](#_Toc412759138)

## 1. Purpose and patterns

The model was designed to illustrate how the eco-evolutionary dynamics of brown trout populations displaying facultative anadromy depend on interactions between sex-specific selection, the extent of pre-existing sexual dimorphism in switch-points (i.e., the threshold value of the energy status trait that controls the migration-residency decision), the presence of frequency dependent selection on male tactics, and genetic architecture. Therefore, the model is suited to simulate changes in the frequency of alternative migratory tactics, via evolutionary responses or phenotypic plasticity, driven by anthropogenic factors. In the present application of the model, we focus on anthropogenic environmental change in the marine compartment driven by increased parasite pressure to address two key questions: (1) how does genetic architecture constrain evolutionary shifts in migration propensity for each sex, and (2) how do associated changes in the intensity of sexual conflict influence population productivity?

To consider our model realistic enough for its purpose, we designed it to reproduce a variety of qualitative patterns described in the literature (see reviews by Jonsson and Jonsson 2006, 2011, Ferguson et al. 2017, 2019, Nevoux et al. 2019), mainly: (1) the benefits and costs of a migratory tactic compared to the other are balanced through their effect on fitness, so relative changes in the mortality rate or growth potential in one environment produces shifts in migration propensity, (2) females tend to predominate amongst migrants while males predominate amongst residents because reproductive success is more dependent on size in females as males can adopt a sneaking mating tactic allowing for successful fertilisation of eggs of large females at a small size, or be a partner of small females through assortative mating, (3) sex-specific optimal switch-points vary with ecological context, and (4) the evolution of sexual dimorphism in switch-points (and hence migration propensity) produces, in some cases, sexual conflict.

## 2. Entities, state variables, and scales

*Scales*. The model’s spatial extent is a square of 100 × 100 square cells (“patches” in NetLogo terminology) of a size defined by the user. The model is spatially explicit and considers two environmental compartments or habitats: the freshwater and marine habitats. Half of the cells belong to each habitat type.

The model runs at a 1-week time step and one simulated year lasts 52 weeks. The duration of the simulation is defined by the user; in this application, each simulation lasts 750 years.

*Entities*. The model has two kinds of entities: (1) **habitat cells** that belong to one of the two habitat types (freshwater/marine); marine cells can be infested with parasites. (2) **Trout** are represented as mobile individuals that are mainly characterized by their location, age, sex, migratory tactic (resident or anadromous), quality, condition, and the genetic material coding for the threshold trait on which the migration-residency decision is based on (Table S1). An individual’s condition represents its energy status (Ferguson et al. 2019) at the temporal window when the decision whether to become anadromous or remain resident is made. We used body length as a proxy for an individual’s quality to make model parameterization easier.

**Table S1.** Entities included in the Sea Trout Model with their state variables and units of measurement.

| **Agent** | **Variable** | **Description** | **Unit** |
| --- | --- | --- | --- |
| Trout | age | Number of weeks since the fish was born | weeks |
|  | sex* | Sex | male/female |
|  | habitat | Compartment where the fish is located | fresh/marine |
|  | state | Health status (whether the fish has parasites or not) | healthy/parasitised |
|  | mother* | Identity of the fish mother | ID |
|  | father* | Identity of the fish father | ID |
|  | z_thresh* | Phenotypic value of the condition threshold trait | unitless |
|  | GM* | Genetic matrix (2 chromosomes with 21 loci coded by 1 or 0) | 21x2 binary matrix |
|  | G* | Genotypic value for the condition threshold trait | unitless |
|  | e_thresh* | Enviornmental component for the condition threshold trait | unitless |
|  | cond* | Condition trait | unitless |
|  | anadromous* | Migratory tactic (whether the fish becomes anadromous) | true/false |
|  | quality | Quality | unitless |
|  | sea-time | Time spent by anadromous fish in the sea since migration | weeks |
|  | mates | Set of mature males selected by a female for reproduction | Agentset |
| Patches | habitat* | Environment | fresh/marine |
|  | parasites?* | Whether the patch has parasites or not | true/false |

* Fixed state variables (do not change during a simulation).

##

## 3. Process overview and scheduling

*Processes*. The model considers four main processes performed by trout: two of them take place on every weekly time step (mortality and aging), while seaward and return migrations, and reproduction occur at one specific week of the year. Habitat cells do not perform any active action. The observer updates time variables, graphical displays and counters, and writes output files.

*Schedule*. All actions occur in the same predetermined order:

1. **Time updates**. The observer updates time variables (*go* NetLogo procedure).
2. **Trout update time variables**. All trout age and trout in the marine habitat update their *sea-time* state variable (*go*).
3. **Trout survive or die**. Senescent trout die and younger individuals challenge their survival against two mortality sources:

3.1. Senescence. All individuals die once they reach the age of maximum longevity, defined by the parameter *lifespan* (*go*). Longevity of brown trout increases with latitude in Europe, the typical lifespan increasing from 5 years or less in Southern Europe to 11 years in the north (Jonsson and Jonsson 2006, 2011). We used an intermediate value of 8 years (416 weeks).

3.2. Density-dependent mortality (*grim-reaper*). If population abundance in the freshwater habitat is over its carrying capacity, the surplus of individuals die.

3.3. Density-independent mortality (*mortality*). Trout are subject to stochastic mortality (e.g. predation, starvation, disease); the probability of surviving varies across habitats.

1. **Anadromous trout migrate**. Anadromous individuals that are ready to migrate move either to the ocean or back to fresh water:

4.1. Migrate to the ocean (*migrate-to-ocean*; week 14). Age-2 and older anadromous trout move to the marine habitat. If the trout arrives at a patch with parasites, its *state* variable is updated.

4.2. Migrate to fresh water (*migrate-to-freshwater*; week 44). Anadromous trout return to the freshwater habitat after spending one winter (first-time spawners) or one summer (repeat spawners) in the sea. The *quality* and *state* (if applicable) state variables are updated.

1. **Sexually mature trout reproduce**. Spawners produce offspring, which inherit from their parents the genetic material coding for the threshold trait that determines the migration-residency decision (week 48):

5.1. Resident spawners change their location in fresh water before reproduction (*go*).

5.2. Resident male spawners try to sneak (*sneaker*). If the sneaker tactic is simulated, resident male spawners sneak when anadromous male spawners predominate in the breeding system.

5.3. Female spawners select mates (*choose-mates*). Female spawners select up to five male spawners with the highest quality to fertilize their eggs.

5.4. Females spawn (*reproduce*). Female spawners produce eggs and each egg is randomly fertilized by one of the previously selected mates.

5.5. Genetic transmission of the heritable trait (*genetic-coding-transmission*). The genetic material (*GM*) of each newly born individual is determined from its parents.

5.6. Migratory tactic is adopted (*set-migratory-behaviour*). Each newly born trout calculates its *condition* and condition threshold *z_thresh* and decides whether it adopts a resident or an anadromous tactic.

1. **Outputs are updated and written**. The observer updates model graphical outputs and counters in the interface. Output files are written.

## 4. Design concepts

*Learning*, *Prediction* and *Collectives* concepts do not apply to this IBM.

*Basic principles*:

The model explores theoretical concepts of ‘partial migration‘, where populations comprise a mix of migratory and non-migratory (resident) individuals (Chapman et al. 2011a). Partial migration typically reflects conditional strategies, whereby individuals of a given genotype facultatively choose migration or residency depending on environmental conditions (Chapman et al. 2011b; Pulido 2011). Facultative migration can be considered as a classic quantitative genetic trait, i.e. controlled by multiple genes and environmental factors, but in this case, the life-history decision is dichotomous with the life history being one of two alternative options controlled by a threshold. Therefore, our model is underpinned by quantitative genetics and is framed conceptually into the ‘environmentally cued threshold model’ (Hazel et al. 1990, Tomkins and Hazel 2007, Buoro et al. 2012), which posits that the decision involves two components: a liability trait or cue–which could be a dynamic physiological variable such as energy status that provides an integrated signal of recent environmental experience to the animal–and a genetically determined threshold for that status or condition, which determines the migration-residency decision.

The model also explores the role of sexual conflict on migration propensity. With intralocus sexual conflict, alleles influencing the same phenotypic trait in each sex have opposing fitness effects on males and females (Lande 1980, Rice 1984). A shared genetic architecture constrains independent evolution of each sex, such that one or both get displaced from their distinct phenotypic optima (Chapman et al. 2003, Bonduriansky and Chenoweth 2009), which reduces the average individual fitness observed within the population (Lande 1980). Modifications to genetic architecture allow males and females to evolve more independently, resulting in increased sexual dimorphism. In consequence, females might be selected to choose migration at a different energy status or condition, for example, compared to males, leading to differential sex-dependent expression of the alternative migratory tactics.

*Emergence*:

The model’s primary result–proportion of anadromous vs. resident individuals–and intermediate results such as trout abundance, productivity (total number of produced eggs) and spatial distribution (freshwater vs. marine habitat) emerge from the growth (represented through *quality*), survival and reproduction of individuals, individual-level processes that are determined by the individuals‘ early decision of whether to migrate to the sea or become resident. This decision in our model is genetically determined (but influenced by the environment), so the trait involved is subject to evolution. The decision to migrate depends on a trade-off between benefits and costs of migration compared with residency, so adaptive evolution should favour strategies that minimize the ratio between mortality rate and growth in quality. Therefore, the proportion of resident relative to migratory fish emerges from variations in growth opportunities and migratory costs (in terms of survival) across the freshwater and marine habitats. Growth opportunities and migratory costs are affected in our model by the presence of parasites in the sea, so the intensity of infestation (number of infested cells), as well as its effects on mortality and growth, influence total trout abundance and proportion of individuals displaying each migratory tactic.

In addition, differential sex-dependent expression of migration and residency can emerge in our model from intralocus sexual antagonism (modelled through the matrix of weights; see *Initialization* and *Reproduction submodel* sections) and the presence of frequency dependent selection on mating tactics of resident male spawners (see *Sneaker submodel* section).

*Adaptation*:

The decision of whether to migrate to the sea or remain in the fresh water is the main adaptive trait in the model. It is modelled as a fitness-seeking process, in which individuals decide early in life a life-history tactic that directly contributes to future success at passing on their genes. This decision affects their future quality (fecundity in females, access to females for males) and survival, and, thus, their fitness. Resident sexually mature males explicitly seek to increase their fitness by adopting a sneaking mating tactic when anadromous male spawners are over-represented in the breeding system.

Selection of male spawners by female spawners is based on the male’s quality, an indirect fitness-seeking behaviour that implicitly assumes to indirectly convey success or fitness as the offspring’s genetic material is inherited from their parents.

*Objectives*:

Through the adoption of the migratory tactic (migration vs. residency), trout seek to balance the potential growth in quality with the probability of surviving until reproduction in order to increase their reproductive success, measured as their lifetime fecundity. If an individual’s condition (i.e., its energy status) is greater than the phenotypic value of the sex-specific condition threshold, it becomes resident, otherwise it becomes anadromous.

Resident sexually mature males aim at increasing their reproductive success by sneaking fertilization of eggs. They become sneakers when the proportion of anadromous males relative to residents within a certain radius (*sneaker_radius*) is greater than the *sneaker_threshold*.

*Sensing*:

Trout are assumed to know their internal state (condition, health status) and the value of all their state variables (sex, age, location, time spent at sea, quality, condition threshold), which influence their migratory tactic, survival, quality, spawning, and movement. Spawners are assumed to perceive the quality of all other spawners within a radius (*female-mate-radius* for female spawners and *sneaker_radius* for resident male spawners).

*Interaction*:

Individuals do not directly interact together, except during reproduction, when sexually mature males indirectly compete for female access through their relative quality. An additional indirect modelled interaction is the adoption by resident males of a sneaking mating tactic when anadromous males are over-represented in the reproductive stock. Density-dependent mortality when the population is over the freshwater carrying capacity is also implemented.

*Stochasticity*:

Stochasticity is used at initialization as the initial location of individuals in the freshwater and marine habitats is randomly assigned and their sex, genetic material for the condition threshold and its phenotypic expression, condition and initial quality are set by drawing values from probability distributions describing their variability. The marine cells that are infested with parasites are also selected randomly.

The most important process represented as totally stochastic is trout mortality: whether a trout dies is determined by a Bernoulli trial using the corresponding mortality rate, and the trout dead through density-dependent mortality are randomly chosen with a probability inversely proportional to their age. Stochasticity is also included in the genetic transmission of the condition threshold trait and the adoption of the migration-resident tactic: first, the male spawner fertilizing a given egg is randomly chosen from the female’s set of mates; second, the new born individual inherits one branch of code from each parent to create its genome, and the allele selected at each locus conforming the branch is randomly chosen; third, a random number (generated from a normal distribution) is added to the genotypic value of the condition threshold trait to define its phenotypic expression; lastly, the actual condition of the individual is drawn from a normal distribution. Newborn individuals move to a random freshwater cell after their creation and their initial quality is also drawn from a normal distribution. Finally, anadromous trout move to a random cell during each migration, so whether a migrant becomes infected with parasites at sea is stochastic.

*Observation*:

The model produces graphical displays and provides output through counters at the interface. They track weekly: number of fish broken out by sex and migratory tactic; number of fish in the marine habitat; number of parasitised fish; mean, variance and SD of *G* of males and females; mean quality of males and females; proportion of each migratory tactic broken out by sex; and number and proportion of anadromous and resident spawners.

Just after reproduction (week 48), if *write-outputs?* switch is on, the model writes four output files in csv format. (1) The “PopulationOutput” file contains yearly population data: total number of mature trout, anadromous spawners, resident spawners, sneakers, eggs fertilized by sneakers, eggs produced and number of newborns, as well as the proportions of anadromous trout, anadromous females, anadromous males, anadromous spawners and resident spawners. (2) The “IndividualOutputSpawners” file collects individual data of each spawner: its sex, age, accumulated fecundity, G value, G value of its mother and its father, mean and variance of the G values of their sons, and mean and variance of the G values of their daughters. (3) The “IndividualOutputNewborns” file collects individual data of each newborn trout: its sex, and the G value and ID of its mother and its father. (4) The “AlleleFreqs” file tracks the frequency of the favourable allele (1) at each locus in the newborn population. The “PopulationOutput” file is always written; the “IndividualOutputSpawners” and “IndividualOutputNewborns” files are written if the *Gcors-outputs?* switch is on (in the case of the newborns file, the *newborns-outputs?* switch has to be activated too); the “AlleleFreqs” file is written if the *track-alleles?* switch is on. The parameter *OutputFreq* defines the frequency (in years) with which output files are written.

## 5. Initialization

The observer time variables are set based on the initial date specified in the *set-environment-procedure*. Our simulations start on December 1 (week 48). In this same procedure, the global environment is initialized as follows: first, the freshwater and marine habitats are created, and then a proportion of marine cells set by the parameter *prop-parasites* become infested with parasites. These cells are selected randomly.

Parameters (see description in Table S2) are set to values specified in the *set-parameters* procedure. The user must indicate whether resident males can display a sneaker tactic (through the *sneaker?* switch in the interface). It must also be specified the number, if any, of loci wherein there is sexual antagonism through the parameter *n-loci-sign*. If sexual antagonism is modelled, the *n-loci-sign* first rows of the 21 x 1 matrix of weights *WMc* for males (see submodel *set-migratory-behaviour*) are multiplied by -1. Thus, unless sexual antagonism is modelled, the matrices of weights for males and females are identical, and are input to the model (see submodel *set-migratory-behaviour* for the description of weight estimation in this application of the model). The mean genotypic value of the condition threshold trait for the male and female populations at initialization (*Gpm* and *Gpf*, respectively) are calculated as the sum of all elements of the matrix of weights *WMc* and *WM*, respectively. Parameters *mu_condM* and *mu_condF* take the same value as *Gpm* and *Gpf*, respectively. The variance of the condition threshold trait *V_cond* is also estimated based on elements of the matrix of weights *WMc* and *WM* (see submodel *set-migratory-behaviour*).

The trout population is initialized in the *set-population* procedure. The initial number of trout is set by the parameter *n-trout*. All trout are initialized as age-2 individuals (*age* = 104 weeks) with a healthy state. Each individual’s state variable (sex, phenotypic and genotypic values of the condition threshold, condition, quality) is initialized by drawing values from probability distributions describing their variability. The genome of each initial fish (described by their genetic matrix *GM*) is composed of a sequence of 21 loci with binary alleles (values 0 or 1) that are randomly assigned with equal probability (=0.5). This formulation guarantees that circa 50% of the male and female populations are anadromous and the other 50% residents. The migratory tactic of initial fish is set as described in the *set-migratory-behaviour* submodel. Resident trout are randomly placed in the freshwater habitat, while anadromous individuals are randomly placed in the marine habitat and initialized with a *sea-time* value of 34, so that they can return to reproduce the next spawning season.

By default, the model is initialized as described above. However, the user has the option to modify the default settings to perform different experiments:

1. A regular simulation starts with an unstructured population comprised only of age-2 trout, but a model run can be initialized with a pre-defined age and life-history population structure obtained from previous simulations through the *start-from-stable-structure* procedure if the *Load-world* button (instead of the *setup* button) is pressed. We used the regular approach (only with age-2 trout) to start our simulations.
2. Individuals can be initialized at their sex-specific optimum switch-point (*start-from-optimum?* switch in the interface is on), which must be specified through the parameters *Gopt-male* and *Gopt-female*. To do this, the probability of the favourable allele (1) being assigned in a given locus is (0.5 x *Gopt-female* / *Gpf*) for females. In the case of males, the probability also depends on *n-loci-sign*: for the (20 - *n-loci-sign*) loci with positive weight, the probability *p* is (0.5 x *Gopt-male* / *Gpm*); for the *n-loci-sign* loci with negative weight, the probability is (1 - *p*).
3. The evolution of the condition threshold trait can be turned off through the *evolution?* switch. If evolution is not modelled, genotypic values of the condition threshold of all trout are set to the sex-specific population mean value at initialization (*Gpm* and *Gpf*). Individual variability in the phenotypic value of the trait is then only driven by environmental variance.

## 6. Input data

The model does not use input data to represent time-varying processes.

## 7. Submodels

7.1. *Density-dependent mortality in fresh water* (*grim-reaper*).

If the population in fresh water is over its carrying capacity, set by the parameter *carryingCapacity*, the surplus of individuals die. Dead trout are randomly chosen, but the probability of each individual being picked is inversely proportional to a weight given by its age. We modelled it using the weighted random drawn method (a “roulette-wheel selection”) implemented in the NetLogo *Rnd* extension.

7.2. *Density-independent mortality* (*mortality*).

Each trout challenges its survival by means of a Bernoulli trial using the mortality rate. The mortality represents the probability of dying during the current week, so it is between 0-1. The mortality rate differs between males (*mortalityM*) and females (modified by the scalar *mortalityF*, which increases/decreases freshwater mortality in females relative to males), freshwater and marine (modified by multipliers *anad-death-multiplierM* and *anad-death-multiplierF*, which are scalars that represent the increased mortality experienced by anadromous fish at sea) habitats, and between healthy and parasitised (increased by multiplier *parasite-load*) individuals. That is, *mortalityM* is the background mortality of males in fresh water, while mortality of females in fresh water is calculated as *mortalityM* x *mortalityF*; the background mortality of males in the marine habitat is *mortalityM* x *anad-death-multiplierM*, and mortality of females in the marine habitat is calculated as (*mortalityM* x *mortalityF*) x (*anad-death-multiplierM* x *anad-death-multiplierF*).

We used the same mortality rate for males and females (*mortalityF* = 1), which was calibrated to reproduce a stable population with a resident/anadromous ratio of 1 (see *check-stability* submodel); the derived value was 0.01543, which represents a 1.5% survival rate between the egg stage and seaward migration (122 weeks). (We assumed that only 10% of the eggs produced per female survive to the next stage; see section 7.7.) Egg-to-smolt mortality rates reported for sea trout are highly variable, but Poole et al. (2006) reported a range between 0.5-3% and Gargan et al. (2006) around 2% for different Irish river systems. Likewise, we used the same mortality rate at sea for males and females (*anad-death-multiplierF* = 1), the value being also calibrated as described before; the estimated value of *anad-death-multiplierM* was 1.262 (so the mortality rate at sea was 0.01944), which represents a 20% survival rate from the smolt stage to the return to fresh water (82 weeks), a value between the 13% reported for northern populations of one sea-winter sea trout by Jonsson and Jonsson (2011) and that reported by Euzenat et al. (2006) for French populations (24%).

Finally, salmon lice-induced mortality of sea trout post-smolts has ranged between 25-46% in experimental conditions (see review by Thorstad et al. 2015), and up to 65% in modelling studies (Hedger et al. 2021). Skaala et al. (2014) reported that the survival rate of tagged smolts from a Norwegian river treated with Substance EX was twice as high as that of untreated smolts. Poole et al. (2006) detected a sea trout population collapse in Western Ireland in 1989 linked to infestations by sea lice from salmon farms, as marine survival of finnock (inmature age-0 trout that spend one summer in the sea) abruptly declined from a 20% historical average to a 6.8% afterwards. Gargan et al. (2006) recorded in other western Ireland catchments a total smolt marine survival between 0.5-5% post-collapse.

7.3. *Seaward migration* (*migrate-to-ocean*).

At week 14 (corresponding to early April), anadromous fish in fresh water older than 104 weeks, i.e. all age-2 (.2+) and older anadromous trout, move to the marine habitat. We selected this life history as most sea trout populations from the UK, Ireland and similar latitudes migrate to sea as smolts at an age of two or three years (Jonsson and Jonsson 2006, Solomon 2006). Migrants are placed in the marine habitat in a random cell, and if the migrant lands in an infested cell, its state changes to “parasitised”.

7.4. *Return migration* (*migrate-to-freshwater*).

At week 44 (corresponding to late October / early November), anadromous fish in the marine habitat that have spent more than 80 weeks there (i.e., through two summers and one winter, 1SW first-time spawners) move to the freshwater habitat, being placed there in a random cell, and reset their *sea-time* state variable. After first reproduction, repeat spawners migrate back to the fresh water to reproduce every year, so they spend only 30 weeks at sea (one summer). This is the typical migration pattern observed in British and Irish populations (Solomon 2006).

Migrating fish update their *quality* state variable by adding the value of the parameter *anad-quality*, which represents the quality gained by the fish by feeding in the sea. In this application of the model, it represents the difference in length growth between the marine and freshwater habitats, not the absolute length growth in the sea. That is, resident trout do not increase their quality, and migrants increase it by the difference in growth across habitats. We set the mean quality of residents (*res_quality_mean*) to 230 (see *set-migratory-behaviour* submodel). Mean length increment of immature brown trout during the first year at sea is between 7 and 22 cm, being similar during the second year (Jonsson and Jonsson 2011). We selected a value for *anad-quality* (200 mm) that falls close to the upper limit of that range. Therefore, first time spawners reproduce at a mean size around 430 mm, within the range reported for .2+1SW sea trout from British and Irish populations (300-460 mm; Butler and Walker 2006, Poole et al. 2006, Solomon 2006). Since repeat spawners migrate back annually, their increase in quality is proportional to the time spent at sea (*anad-quality* x 30 / 82). Thus, individuals can migrate more than once and accrue additional quality benefits, but the quality gained depends on the time spent at sea. No additional mortality costs were assumed for these repeat spawners.

If the individual is infected with parasites (*state* = “parasitised”) then the quality gained by being in the sea is reduced by a factor, defined by parameter *paras_quality*. Therefore, the increase in quality for parasitised fish is *anad-quality* x *paras_quality*. Fjørtoft et al. (2014) reported a reduction in sea trout body mass growth of 20-40%, potentially linked to salmon lice spread from a local salmon farm; Birkeland (1996) found a median decrease of 23.5% in body mass among sea trout delousing during five weeks in fresh water before returning to the sea, but modelling studies predict that the reduction in growth potential by a 6-week delousing stay in fresh water can be up to 59% (Halttunen et al. 2018). Once parasitised fish return to fresh water, they are assumed to lose all their lice–which do not survive in freshwater (Skaala et al. 2014)–, and do not experience any further parasite effects on mortality or quality.

7.5. *Adopt a sneaking tactic* (*sneaker*).

In wild populations, resident males can adopt a mating tactic by which they evade competition and aggression from large anadromous males by sneaking fertilisations (Gross 1985). This tactic is likely to be under negative frequency dependent selection, where sneakers have higher fitness only when rare (Gross 1996, Taborsky et al. 2008). Therefore, in our implementation, resident males search in a radius set by parameter *sneaker_radius* (20 cells in our model application; the same value as the *female-mate-radius*) and count the proportion of anadromous males relative to residents. If the proportion of anadromous males is greater than the *sneaker_threshold* (0.7 in this study), then the resident male temporarily gets a boost to its quality, defined by the parameter *sneaker_boost*. We set *sneaker_boost* to a value (500) much higher than the quality gained by healthy anadromous individuals in the marine habitat (Young et al. 2013). After reproduction is over, the sneaker resets its quality to its initial value (*start_quality* state variable).

7.6. *Selection of male mates by females* (*choose-mates*).

Trout are sexually mature in their second year of life (*age* > 104 weeks). Thus, resident trout are mature in their third year of life (age-2 trout), while anadromous individuals start to reproduce in their fourth year of life after spending one winter at sea (age-3), reflecting the fact that age at sexual maturity is typically lower in resident than anadromous trout in wild populations (Jonsson and Jonsson 2006). At week 48 (corresponding to late November/early December), each female spawner in the fresh water selects the five mature males with the highest quality within a radius defined by the parameter *female-mate-radius* (20 cells in our model application). Selected males comprise the *mates* set of the female spawner.

7.7. *Reproduction* (*reproduce*).

Female spawners produce a number of eggs that depends on the the spawner’s fecundity. Fecundity is modelled as a function of quality through a power function:

*fecundity* = *a quality^b^*

where *a* and *b* are model parameters. We parameterized this function based on the length-fecundity relationship developed by Solomon (1997) for sea trout populations in the British Isles: Log_10_ *F* = 2.7514 Log_10_ *L* – 4.0623.

Each egg is fertilized by a male randomly chosen from the female’s mates, with equal probability of selection across mates, with the selected mate becoming the father of the newborn trout. The number of eggs produced by the female was translated into number of fry by assuming that only a proportion of the eggs, set by parameter *SurvRate*, survived to the next stage. We assumed that only 10% of eggs survive through egg development and the critical period after emergence (Elliott 1994). Accumulated fecundity *FecAcc* of the female spawner is then updated.

7.8. *Genetic transmission of the heritable trait*.

If after reproduction the population in the fresh water is over its carrying capacity, the surplus of newborn individuals die, the dead individuals being randomly chosen. Thus, genetic transmission is only modelled in surviving individuals.

The model includes a diploid, bi-allelic, multilocus system for the condition threshold trait. For the trait, two branches representing two chromosomes are attributed with 21 loci, where each locus can take values of either 1 or 0. The last locus is a neutral marker and has no genetic effect. The genome of each fish is represented by a 21 x 2 matrix (*GM*), in which each column represents one branch and each row a locus.

The genome of a newborn trout is determined from the genetic material of its respective parents. One of the two alleles (from the two branches) at each locus is randomly chosen to make up a unique branch of code per parent. The two branches from the two parents are then transferred to the new individual to create its *GM*.

7.9. *Set migratory tactic and initial quality* (*set-migratory-behaviour*).

*Set migratory tactic*:

The phenotype of the condition threshold *z_thresh* is defined as the sum of its additive genotypic value *G* and a statistically independent, random environmental effect *e_thresh* drawn from a normal distribution N(0, *Ve*):

*z_thresh* = *G* + *e_thresh*

If evolution is modelled, males and females get the genotypic value for the threshold trait according to their genome, represented by the genetic matrix *GM*. The contribution of each locus to the genotypic value of the condition threshold trait is defined by the sex-specific matrix of weights (*WM* for females, *WMc* for males), a 21 x 1 matrix in which each row is the weight of the given locus. The user can choose whether all loci have equal weights or values are estimated from a negative exponential function (switch *loci-equal-weighting?* in the interface on vs. off). If the former, all weights have an equal absolute value of 0.5. If the latter, weights are estimated according to a negative exponential function so that their sum equals the population mean of the additive genotypic value at initialization (set to 10). This is calculated by solving for lambda (λ) in the following equation:

$\sum_{i=1}^{20} e^{-\lambda i}$= 10

The genotypic value *G* of an individual is then computed by matrix multiplication:

*G* = *sum-G-matrix* x *WM*

where *sum-G-matrix* is a 1 x 21 matrix resulting from the matrix addition of the first and second columns (branches) of *GM* and subsequent transposition. *WM* is the matrix of weights (*WMc* for males).

We calculated the additive genetic variance *Va* of the condition threshold trait as the expectation of the variance for the sum of independent weighted Bernoulli random variables:

*Va* = *p* x (1 – *p*) x $\sum_{i=1}^{20} w_{i}^{2}$

where each random variable (locus) takes the value 1 with probability *p* (0.5) and 0 with probability (1 – *p*) and *w_i_* is the weight of each locus.

Since estimates of narrow-sense heritability (*h^2^*; i.e., the additive genetic portion of the phenotypic variance) for anadromy in salmonids is around 0.5 (see Ferguson et al. 2019 and references therein), we assumed that half of the phenotypic variability in the condition threshold at the population level is due to additive genetic variance and half to environmental influences. Therefore, we set the variance of the environmental effect *Ve* equal to *Va*.

If evolution is not modelled, the *G* value for each newly born individual is set to the sex-specific population mean value at initialization (either *Gpm* or *Gpf*).

The value of the condition trait *cond* is drawn from a normal distribution with mean *mu_cond* and variance *V_cond*. The parameter *mu_cond* is sex-specific and was set equal to the population mean phenotypic value of the sex-specific condition threshold trait at initialization (mean *z_thresh* = *Gpm* or *Gpf* ). Since all the phenotypic variance of the condition trait is attributed to environmental conditions, *V_cond* was set equal to *Ve*. The condition trait *cond* is then compared to the phenotypic value of the condition threshold *z_thresh* and if an individual’s condition is greater than its threshold value, it becomes resident (i.e., *anadromous* = false), otherwise it becomes anadromous (*anadromous* = true).

*Set initial quality*:

Individuals get the initial value of their *quality* state variable (*start_quality*) from a normal distribution with mean *res_quality_mean* and standard deviation *res_quality_sd*. We set *res_quality_mean* to 230, a value in the middle of the ranges reported for age-2 resident spawners in UK rivers by Elliott (1984) (180-300 mm) and Solomon (2006) (180-286 mm), and in the upper limit of the range reported for Norwegian populations by Jonsson and Jonsson (2011) (152-230 mm). We set *res_quality_sd* to 5% of *res_quality_mean*.

**Table S2.** Model parameter descriptions and selected values with their sources. Units are in parentheses.

| **Parameter** | **Description** | **Value** | **Source** |
| --- | --- | --- | --- |
| *n-trout* | Number of trout at initialization (#) | 3000 | - |
| *prop-parasites* | Proportion of marine patches infested with parasites (0-1) | Experiment | - |
| *lifespan* | Maximum trout longevity (weeks) | 416 | Jonsson & Jonsson (2011) |
| *carryingCapacity* | Carrying capacity of the freshwater habitat (#) | 3000 | - |
| *mortalityM* | Mortality rate of males in the freshwater habitat (probability of dying during a week; 0-1) | 0.01543 | Calibrated |
| *mortalityF* | Extra mortality for females in the freshwater habitat realtive to males (multiplier) | 1 | - |
| *anad-death-multiplierM* | Extra mortality for males in the marine habitat relative to the fresh water habitat (multiplier) | 1.262 | Calibrated |
| *anad-death-multiplierF* | Extra mortality for females in the marine habitat realtive to males (multiplier) | 1 | - |
| *parasite-load* | Extra mortality due to parasites (multiplier) | Experiment | - |
| *sneaker?* | Indicates whether resident males use a sneaker tactic (Boolean) | Yes | Ferguson et al. (2019) |
| *sneaker_radius* | Radius within which mature resident males count the number of competing mature anadromous males (cells) | 20 | Guesstimated |
| *sneaker_threshold* | Proportion of mature anadromous males inside the *sneaker_radius* over which a mature resident male becomes a sneaker (0-1) | 0.7 | Guesstimated |
| *sneaker_boost* | Increase in quality experienced by sneakers during reproduction | 500 | Guesstimated |
| *female-mate-radius* | Radius within which mature females select mature males for reproduction (cells) | 20 | Guesstimated |
| *a* | Scaling factor of the fecundity power function | 0.0000866 | Solomon (1997) |
| *b* | Power factor of the fecundity power function | 2.7514 | Solomon (1997) |
| *SurvRate* | Proportion of trout that survive the critical period after emergence (0-1) | 0.1 | Elliott (1994) |
| *Ve* | Variance of the normal distribution used to define the environmental effect on the condition threshold trait | 2.94706 | Theoretical |
| *WM* | Matrix of weights defining the contribution of each locus to the genotypic value of the condition threshold *G* of females | Input matrix | Theoretical |
| *WMc* | Matrix of weights defining the contribution of each locus to the genotypic value of the condition threshold *G* of males | Experiment | - |
| *n-loci-sign* | Number of loci that have a different sign in the males’ matrix of weights *WM* (0-20) | Experiment | - |
| *Gpm* | Mean genotypic value of the condition threshold *G* of the male subpopulation at initialization | Depends on *WMc* | Theoretical |
| *Gpf* | Mean genotypic value of the condition threshold *G* of the female subpopulation at initialization | Depends on *WM* | Theoretical |
| *mu_condM* | Mean of the normal distribution used to define male trout condition | Depends on *WMc* | Theoretical |
| *mu_condF* | Mean of the normal distribution used to define female trout condition | Depends on *WM* | Theoretical |
| *V_cond* | Variance of the normal distribution used to define trout condition | 2.94706 | Theoretical |
| *res_quality_mean* | Mean of the normal distribution used to define the initial quality of trout in fresh water (mm) | 230 | Elliott (2004), Solomon (2006) |
| *res_quality_sd* | Standard deviation of the normal distribution used to define the initial quality of trout in fresh water (mm) | 20 | Guesstimated |
| *anad _quality* | Increase in quality that anadromous trout experience every time they migrate to the sea (mm) | 200 | Jonsson & Jonsson (2011) |
| *paras_quality* | Decrease in the quality gained in the sea by anadromous trout due to parasites (proportion; 0-1) | Experiment | - |

7.10. *Check-stability*.

This submodel is used to check whether the modelled population has reached a stable structure in which half of the population presents an anadromous migratory tactic and half are residents. To do this, every year (just after reproduction, at week 48) after an initial warm-up period (set by the *Burnin* parameter), the submodel checks whether the stability criteria are met, and if so, the simulation ends and the current simulation state is saved into a csv file, whose path is defined in the *exp-world-filepath* parameter. This file can be imported at initialization to define the initial population structure (see Initialization section). We defined three stability criteria: (1) the proportion of anadromous trout in the population (*prop-anad*) in the current year is within *mean-anad* x (1 ± *var-mean-anad*), (2) the coefficient of variation of *prop-anad* over a period of time defined by the parameter *stability-period* is below a defined threshold (*Cv-thr-anad*), and (3) the mean value of *prop-anad* over the *stability-period* is within the range *mean-anad* x (1 ± *Cv-thr-anad*/2). These stability criteria (with *mean-anad* =0.5, *var-mean-anad*=1%, and *Cv-thr-anad*=4%) were used to calibrate the values of *mortalityM* and *anad-death-multiplierM*, i.e. we chose the value of these parameters that led to a stable population structure.

## 8. References

Birkeland, K. (1996) Consequences of premature return by sea trout (*Salmo trutta*) infested with the salmon louse (*Lepeophtheirus salmonis* Krøyer): migration, growth, and mortality. Canadian Journal of Fisheries and Aquatic Sciences 53(12), 2808-2813.

Bonduriansky, R. & Chenoweth, S. F. (2009) Intralocus Sexual Conflict. Trends in Ecology & Evolution 24, 280-288.

Buoro, M., Gimenez, O. & Prévost, E. (2012) Assessing Adaptive Phenotypic Plasticity by Means of Conditional Strategies from Empirical Data: The Latent Environmental Threshold Model. Evolution: International Journal of Organic Evolution 66, 996-1009.

Butler, J. R. A., & Walker, A. F. (2006) Characteristics of the sea trout *Salmo trutta* (L.) stock collapse in the River Ewe (Wester Ross, Scotland), in 1988-2001. In: Sea Trout: Biology, Conservation and Management (Harris, G., Milner, N., eds), pp. 45-59. Blackwell Publishing Ltd, Oxford, UK.

Chapman, T., Arnqvist, G., Bangham, J. & Rowe, L. (2003) Sexual Conflict. Trends in Ecology & Evolution 18, 41-47.

Chapman, B. B., Brönmark, C., Nilsson, J.-Å. & Hansson, L.-A. (2011a) Partial Migration: An Introduction. Oikos 120, 1761-1763.

Chapman, B. B., Brönmark, C., Nilsson, J.-Å. & Hansson, L.-A. (2011b) The Ecology and Evolution of Partial Migration. Oikos 120, 1764-1775.

Elliott, J.M. (1984) Numerical changes and population regulation in young migratory trout *Salmo trutta* in a lake district stream, 1966-83. Journal of Animal Ecology 53, 327-350.

Elliott, J.M. (1994) Quantitative Ecology and the Brown Trout, Oxford University Press, UK.

Euzenat, G., Fournel, F., & Fagard, J. L. (2006) Population dynamics and stock–recruitment relationship of sea trout in the River Bresle, upper Normandy, France. In: Sea Trout: Biology, Conservation and Management (Harris, G., Milner, N., eds), pp. 307-323. Blackwell Publishing Ltd, Oxford, UK.

Ferguson, A., Reed, T., McGinnity, P., & Prodöhl, P. (2017) Anadromy in brown trout (*Salmo trutta*): A review of the relative roles of genes and environmental factors and the implications for management and conservation. In: G. Harris (Ed.), Sea trout: Management & science (Harris, G., ed), pp. 1-40. Matador Publishing Ltd, Leicestershire, UK.

Ferguson, A., Reed, T.E., Cross, T.F., Mcginnity, P. & Prodöhl, P.A. (2019) Anadromy, potamodromy and residency in brown trout Salmo trutta: the role of genes and the environment. Journal of Fish Biology 95(3), 692-718.

Fjørtoft, H. B., Borgstrøm, R., & Skaala, Ø. (2014) Differential changes in growth patterns of anadromous brown trout and Atlantic salmon from the River Etneelva over a 25-year period. Marine Biology Research 10(3), 301-307.

Gargan, P. G., Roche, W. K., Forde, G. P., & Ferguson, A. (2006) Characteristics of the sea trout (*Salmo trutta* L.) stocks from the Owengowla and Invermore fisheries, Connemara, Western Ireland, and recent trends in marine survival. In: Sea Trout: Biology, Conservation and Management (Harris, G., Milner, N., eds), pp. 60-75. Blackwell Publishing Ltd, Oxford, UK.

Gross, M. R. (1985) Disruptive Selection for Alternative Life Histories in Salmon. Nature 313, 47.

Gross, M. R. (1996) Alternative Reproductive Strategies and Tactics: Diversity within Sexes. Trends in Ecology & Evolution 11, 92-98.

Halttunen, E., Gjelland, K.-O., Hamel, S., Serra-Llinares, R.M., Nilsen, R., Arechavala-Lopez, P., Skarohamar, J., Johnsen, I.A., Asplin, L., Karlsen, O., Bjorn, P.A. & Finstad, B. (2018) Sea trout adapt their migratory behaviour in response to high salmon lice concentrations. Journal of Fish Diseases 41, 953-967.

Hazel, W. N., Smock, R. & Johnson, M. D. (1990) A Polygenic Model for the Evolution and Maintenance of Conditional Strategies. Proceedings of the Royal Society of London. Series B: Biological Sciences 242, 181-187.

Hedger, R.D., Diserud, O.H., Finstad, B., Jensen, A.J., Hendrichsen, D.K., Ugedal, O. & Næsje, T.F. (2021) Modeling salmon lice effects on sea trout population dynamics using an individual-based approach. Aquaculture Environment Interactions 13, 145-163.

Jonsson, B. & Jonsson, N. (2006) Life history of the anadromous trout *Salmo trutta*. In: Sea Trout: Biology, Conservation and Management (Harris, G., Milner, N., eds), pp. 196-223. Blackwell Publishing Ltd, Oxford, UK.

Jonsson, B. & Jonsson, N. (2011) Ecology of Atlantic Salmon and Brown Trout: Habitat as a Template for Life Histories. Fish and Fisheries Series. Springer, Dordrecht, Netherlands.

Lande, R. (1980) Sexual Dimorphism, Sexual Selection, and Adaptation in Polygenic Characters. Evolution 34, 292305.

Nevoux, M., Finstad, B., Davidsen, J.G., Finlay, R., Josset, Q., Poole, R., Höjesjö, J., Aarestrup, K., Persson, L., Tolvanen, O. & Jonsson, B. (2019) Environmental influences on life history strategies in partially anadromous brown trout (*Salmo trutta*, Salmonidae). Fish and Fisheries, 20(6), 1051-1082.

Poole, W. R., Dillane, M., DeEyto, E., Rogan, G., McGinnity, P. & Whelan, K. (2006) Characteristics of the Burrishoole Sea Trout Population: Census, Marine Survival, Enhancement and Stock-Recruitment Relationship, 1971-2003. In: Sea Trout: Biology, Conservation and Management (Harris, G., Milner, N., eds), pp. 279–306. Blackwell Publishing Ltd, Oxford, UK.

Pulido, F. (2011) Evolutionary Genetics of Partial Migration – the Threshold Model of Migration Revis(It)Ed. Oikos 120, 1776-1783.

Rice, W. R. (1984) Sex Chromosomes and the Evolution of Sexual Dimorphism. Evolution 38, 735-742.

Skaala, Ø., Kålås, S. & Borgstrøm, R. (2014) Evidence of salmon lice-induced mortality of anadromous brown trout (*Salmo trutta*) in the Hardangerfjord, Norway. Marine Biology Research 10, 279-288.

Solomon, D. J. (1997) Review of sea trout fecundity. R&D Technical Report W60. Environment Agency, Bristol, UK.

Solomon, D. J. (2006) Migration as a life-history strategy for the sea trout. In: Sea Trout: Biology, Conservation and Management (Harris, G., Milner, N., eds), pp. 224-233. Blackwell Publishing Ltd, Oxford, UK.

Taborsky, M., Oliveira, R. F. & Brockmann, H. J. (2008) The Evolution of Alternative Reproductive Tactics: Concepts and Questions. Alternative reproductive tactics: an integrative approach 1, 21.

Thorstad, E. B., Todd, C. D., Uglem, I., Bjørn, P. A., Gargan, P. G., Vollset, K. W., Halttunen, E., Kålås, S., Berg, M. & Finstad, B. (2015) Effects of Salmon Lice *Lepeophtheirus salmonis* on Wild Sea Trout *Salmo trutta* a Literature Review. Aquaculture Environment Interactions 7, 91-113.

Tomkins, J. L. & Hazel, W. (2007) The Status of the Conditional Evolutionarily Stable Strategy. Trends in Ecology & Evolution 22, 522-528.

Young, B., Conti, D. V., & Dean, M. D. (2013). Sneaker “jack” males outcompete dominant “hooknose” males under sperm competition in Chinook salmon (*Oncorhynchus tshawytscha*). Ecology and Evolution, 3(15), 4987-4997.
