# Supplementary material for: Escalating the conflict? Intersex genetic correlations influence adaptation to environmental change in facultatively migratory populations
Source: Evol Appl. 2022 Mar 30;15(5):773–89. doi: 10.1111/eva.13368 (PMC9108303; doi:10.1111/eva.13368)
Supplement: Supplementary file 3 — Appendix S2 [file EVA-15-773-s001.docx]

**Supplementary material:**

**Sensitivity analysis**

We conducted a global sensitivity analysis in order to screen non-influential and influential parameters on the variability of relevant model outputs. The sensitivity analysis examined 11 model outputs measured at the population level: total population abundance (*CountTotalPop*), carrying capacity saturation (i.e., ratio between population abundance in the fresh water to its carrying capacity; *D/K ratio*), total number of anadromous spawners (*CountSpawnAnad*), total number of resident spawners (*CountSpawnResid*), total number of sneakers (*CountSneakers*), total production of eggs in the spawning season (*CountEggs*), number of eggs fertilized by sneakers in the spawning season (*CountSneakerEgg*s), total number of newborn individuals that incorporate into the population (*CountNewborns*), and proportion of anadromous males (*PropAnadMale*), females (*PropAnadFemale*) and spawners (*PropSpawnAnad*). Simulations were run for 750 years and one measure of each model output was recorded each simulated year (after the spawning season was over; week 48) and used to compute a mean for the last 100 years of the model run.

We selected 15 parameters to conduct the global sensitivity analysis (Table B1). We did not include any parameter related to parasite infection (*prop-parasites*, *parasite-load*, and *paras_quality*), since their effects were analyzed in the parasites scenarios. In each model run, the population was initialized at the tested carrying capacity. The matrix of weights for females (*WM*) was set so that all loci had equal weights. All parameters that are calculated from WM (*Gpm*, *Gpm*, *mu_condM*, *mu_condM*, *V_cond* and *Ve*) were logically not included in the analysis.

We used an improved version of the Morris’s elementary effects screening method (Morris 1991; Campolongo et al. 2007) to identify the parameters to which the sea trout model was particularly insensitive or sensitive. This method appears to be the most suited screening method for IBMs (Thiele et al. 2014, Ligmann-Zielinska et al. 2020). Screening methods rank-order input parameters based on their importance and therefore weed out the non-influential inputs. Based on individually randomised one-factor-at-a-time designs, it estimates the effects of changes in the input factor levels, i.e., the parameter values, which are called elementary effects (EEs). The EEs are then statistically analysed to measure their relative importance. We used the estimate of the mean of the distribution of the absolute values of the elementary effects, *μ*^*^, as a sensitivity measure to establish the relative influence of each parameter. It can be considered as a proxy of the total sensitivity index, which itself is a measure of the overall effect of a parameter on the output, including interactions with the rest of the model (Saltelli et al. 2008). All 15 selected parameters (*K*) were varied over five levels according to predefined ranges around the value used to calibrate the model (Table B1). Parameters were varied within observed or biologically plausible (for guesstimated parameters) values. The number of tested settings is given by *r* × (*k* + 1), where *r* is the number of EEs computed per parameter. As we chose 100 EEs, this led to 100 × (15 + 1) = 1600 model runs. We replicated the analysis five times and got an average value of *μ*^*^. We used the *sensitivity* R package (Iooss et al. 2020) to both generate the design of experiments and estimate the sensitivity measures.

**Table B1.** Model parameter descriptions, default values and range used to conduct the global sensitivity analysis.

| **Parameter** | **Description** | **Default value** | **Tested range** |
| --- | --- | --- | --- |
| *lifespan* | Maximum trout longevity (weeks) | 416 | 260-572 |
| *carryingCapacity* | Carrying capacity of the freshwater habitat (#) | 3000 | 1000-5000 |
| *mortalityM* | Mortality rate of males in the freshwater habitat (probability of dying during a week; 0-1) | 0.01543 | 0.00982-0.02426 |
| *mortalityF* | Extra mortality for females in the freshwater habitat realtive to males (multiplier) | 1 | 0.7-1.3 |
| *anad-death-multiplierM* | Extra mortality for males in the marine habitat relative to the fresh water habitat (multiplier) | 1.262 | 0.8-1.8 |
| *anad-death-multiplierF* | Extra mortality for females in the marine habitat realtive to males (multiplier) | 1 | 0.7-1.3 |
| *sneaker_radius* | Radius within which mature resident males count the number of competing mature anadromous males (cells) | 20 | 5-35 |
| *sneaker_threshold* | Proportion of mature anadromous males inside the *sneaker_radius* over which a mature resident male becomes a sneaker (0-1) | 0.7 | 0.6-0.9 |
| *sneaker_boost* | Increased in quality experienced by sneakers during reproduction | 500 | 300-900 |
| *female-mate-radius* | Radius within which mature females select mature males for reproduction (cells) | 20 | 5-35 |
| *b* | Power factor of the fecundity power function | 2.7514 | 2.4763-3.0266 |
| *SurvRate* | Proportion of trout that survive the critical period after emergence (0-1) | 0.1 | 0.05-0.3 |
| *n-loci-sign* | Number of loci that have a different sign in the males’ matrix of weights *WM* (0-20) | 0 | 0-20 |
| *res_quality_mean* | Mean of the normal distribution used to define the initial quality of tout in the fresh water (mm) | 230 | 150-300 |
| *anad _quality* | Increase in quality that anadromous trout experience every time they migrate to the sea (mm) | 200 | 70-300 |

The results from the screening analysis indicated that variations in the proportion of individuals adopting an anadromous life history are mainly driven by changes in male and female mortality rates at both freshwater and marine habitats (Table B2). In the case of males, it is also highly determined by the parameter controlling the degree of sexual antagonism (*n-loci-sign*). In the case of females, the decision of whether becoming migrant is additionally influenced by the increase in individual fitness provided by a higher quality gained at sea (*anad _quality*) and a higher scaling rate of individual fecundity with quality (*b*) (Table B2).

Regarding the sneaker behaviour, the number of sneakers is primarily driven by the parameters that determine the proportion of male spawners adopting an anadromous life history (*mortalityM*, *anad-death-multiplierM* and *n-loci-sign*) and in second place by parameters controlling the interaction of resident and anadromous males (*sneaker_radius* and *sneaker_threshold*) (Table B2). This pattern was expected as the latter parameters can have an effect on triggering sneaker behaviour only when the male population is dominated by migratory individuals. The number of eggs fertilized by sneakers is influenced by parameters defining the number of sneakers and female fecundity.

**Table B2.** Complete sensitivity results as a percentage of the maximum sensitivity (computed as the *μ*^*^ index) for six model outputs related to the spawner subpopulation and the proportion of anadromous individuals within the spawner, male and female subpopulations: total number of anadromous spawners (*CountSpawnAnad*), total number of resident spawners (*CountSpawnResid*), total number of sneakers (*CountSneakers*), and proportion of anadromous males (*PropAnadMale*), females (*PropAnadFemale*) and spawners (*PropSpawnAnad*). Sensitivity values over 75% of the maximum are highlighted in bold.

| **Parameter** | **CountSpawnAnad** | **CountSpawnResid** | **Count Sneakers** | **PropSpawn Anad** | **PropAnadMale** | **PropAnad Female** |
| --- | --- | --- | --- | --- | --- | --- |
| *lifespan* | 27.0 | 31.6 | 53.6 | 50.6 | 58.1 | 51.0 |
| *carryingCapacity* | 53.8 | 62.3 | 43.2 | 39.5 | 56.0 | 39.3 |
| *mortalityM* | **100.0** | **100.0** | **94.6** | **100.0** | **100.0** | **100.0** |
| *mortalityF* | 48.3 | 39.7 | 66.3 | **77.6** | 72.7 | 65.0 |
| *anad-death-multiplierM* | 59.0 | 38.5 | **100.0** | **83.5** | **75.2** | **78.1** |
| *anad-death-multiplierF* | 32.7 | 33.0 | 38.5 | 51.1 | 50.7 | 57.1 |
| *sneaker_radius* | 12.5 | 22.3 | 60.1 | 31.5 | 39.0 | 32.6 |
| *sneaker_threshold* | 9.6 | 17.8 | 65.5 | 27.5 | 41.3 | 21.4 |
| *sneaker_boost* | 10.1 | 20.6 | 54.3 | 25.6 | 35.9 | 33.1 |
| *female-mate-radius* | 20.4 | 35.3 | 53.1 | 45.7 | 66.2 | 32.7 |
| *b* | 15.3 | 38.9 | 43.0 | 65.5 | 54.7 | 64.6 |
| *SurvRate* | 13.7 | 34.2 | 49.5 | 46.5 | 56.9 | 44.5 |
| *n-loci-sign* | 22.4 | 44.2 | **80.8** | 64.8 | **96.8** | 43.0 |
| *res_quality_mean* | 16.0 | 35.7 | 62.8 | 48.7 | 61.6 | 44.5 |
| *anad _quality* | 19.7 | 43.6 | 72.4 | 48.3 | 48.4 | 64.1 |

The results from the screening analysis showed that the vast majority of parameters had little effect on population productivity measures (Table B3). The *carryingCapacity* of the fresh water logically controlled total population abundance and recruitment, but the mortality rate in the freshwater habitat (*mortalityM*) and the fecundity parameter *b* exerted the strongest influence on carrying capacity saturation and total production of eggs. Very high values of *mortalityM* (over 0.02) typically led to population extinction, and only within adequate values of this parameter, an extra/reduced mortality on females (*mortalityF*) had an effect on population numbers and egg production. The quality gained at sea by anadromous female spawners (*anad _quality*) also had a relatively high effect on egg production because of the allometric relationship between quality and individual fecundity. Thus, while background mortality rates at both freshwater and marine habitats controlled the total number of spawners (Table B2), sensitivity analyses indicate that, even if the number of spawners is reduced, they could still produce a high number of eggs if the scaling rate of individual fecundity with quality and the quality gained at sea by migrants are very high. In consequence, the proportion of anadromous female spawners can be high even at high mortality rates at sea when migrating to the sea provides a strong increase in individual fitness (Tables B2 and B3).

**Table B3.** Complete sensitivity results as a percentage of the maximum sensitivity (computed as the *μ*^*^ index) for five model outputs related to population productiviy: total population abundance (*CountTotalPop*), carrying capacity saturation (*D/K ratio*), total production of eggs in the spawning season (*CountEggs*), number of eggs fertilized by sneakers in the spawning season (*CountSneakerEgg*s), and total number of newborn individuals that incorporate into the population (*CountNewborns*). Sensitivity values over 75% of the maximum are highlighted in bold.

| **Parameter** | **CountTotalPop** | **D/K ratio** | **CountEggs** | **CountSneakerEggs** | **CountNewborns** |
| --- | --- | --- | --- | --- | --- |
| *lifespan* | 12.2 | 20.7 | 19.6 | 30.9 | 14.3 |
| *carryingCapacity* | **100.0** | 14.2 | 41.0 | 47.9 | **100.0** |
| *mortalityM* | **75.4** | **100.0** | **100.0** | **96.1** | **96.0** |
| *mortalityF* | 33.4 | 50.4 | 63.2 | 33.6 | 46.0 |
| *anad-death-multiplierM* | 19.5 | 31.8 | 36.9 | 29.1 | 22.5 |
| *anad-death-multiplierF* | 13.1 | 20.0 | 30.5 | 15.0 | 16.2 |
| *sneaker_radius* | 3.2 | 6.0 | 4.0 | 48.6 | 3.9 |
| *sneaker_threshold* | 3.9 | 6.1 | 3.2 | 34.6 | 4.6 |
| *sneaker_boost* | 2.0 | 3.8 | 2.6 | 32.8 | 2.7 |
| *female-mate-radius* | 7.9 | 11.7 | 4.0 | 23.0 | 9.5 |
| *b* | 39.6 | 62.9 | **86.9** | 33.1 | 46.4 |
| *SurvRate* | 21.2 | 29.7 | 44.9 | 34.9 | 24.0 |
| *n-loci-sign* | 7.4 | 12.8 | 3.9 | **75.3** | 8.9 |
| *res_quality_mean* | 19.2 | 26.8 | 25.8 | 37.3 | 22.3 |
| *anad _quality* | 9.2 | 13.1 | 64.6 | **100.0** | 10.5 |

All in all, there are four kinds of parameters strongly affecting both the proportion of anadromous individuals in the population and total population productivity: background mortality rates (*mortalityM* and *anad-death-multiplierM*), the scaling rate of individual fecundity with quality (*b*) and the quality gained at sea by migrants (*anad _quality*). Nevertheless, they are not uncertain parameters since there is a vast number of studies providing reliable values for their parameterization. Conversely, the extra/reduced mortality on females at either habitat (*mortalityF* and *anad-death-multiplierF*) can also have a strong influence on analyzed model outputs but their values are highly uncertain because of paucity in studies addressing this topic. In the simulations presented in the main manuscript, we have adopted a value of 1 in both parameters. The parameter *carryingCapacity* has only strong effects on total absolute numbers and it is a parameter set by the user. The parameter *n-loci-sign* is a main driver of the proportion of anadromous males in the population so its effects on the eco-evolutionary trajectory of the population were explored through simulation scenarios in the main manuscript. Finally, the parameters *sneaker_radius* and *sneaker_threshold* influenced whether the sneaker behaviour is triggered or not and their values are uncertain, so we performed a preliminary local analysis on both parameters to choose adequate values (those indicated on Table B1).

# References

Campolongo, F., Cariboni, J., Saltelli, A. 2007. An effective screening design for sensitivity analysis of large models. Environ. Mod. Softw. 22, 1509-1518.

Ligmann-Zielinska, A., Siebers, P.-O., Magliocca, N., Parker, D.C., Grimm, V., Du, J., Cenek, M., Radchuk, V., Arbab, N.N., Li, S., Berger, U., Paudel, R., Robinson, D.T., Jankowski, P., An, L., Ye, X. 2020. "One Size Does Not Fit All": A Roadmap of Purpose-Driven Mixed-Method Pathways for Sensitivity Analysis of Agent-Based Models. J. Artif. Soc. Soc. Simulat. 23, 6.

Morris, M.D. 1991. Factorial sampling plans for preliminary computational experiments. Technometrics 33, 161-174.

Iooss, B., Da Veiga, S., Janon, A, Pujol, G.. 2020. Sensitivity: Global Sensitivity Analysis of Model Outputs. R Package Version 1.22.1.

Saltelli, A., Ratto, M., Andres, T., Campolongo, F., Cariboni, J., Gatelli, D., Saisana, M., Tarantola, S. 2008. Global Sensitivity Analysis, The Primer. Wiley, Chichester, UK.

Thiele, J.C., Kurth, W., Grimm, V. 2014. Facilitating Parameter Estimation and Sensitivity Analysis of Agent-Based Models: A Cookbook Using NetLogo and 'R'. J. Artif. Soc. Soc. Simulat. 17, 11.
